# Machine Learning and Causal Approaches to Predict Readmissions and Its Economic Consequences Among Canadian Patients With Heart Disease: Retrospective Study

**DOI:** 10.2196/41725

**Published:** 2023-05-26

**Authors:** Ethan Rajkumar, Kevin Nguyen, Sandra Radic, Jubelle Paa, Qiyang Geng

**Affiliations:** 1 Department of Chemistry, Faculty of Science The University of British Columbia Vancouver, BC Canada; 2 Department of Computer Science, Faculty of Science The University of British Columbia Vancouver, BC Canada; 3 School of Biomedical Engineering, Faculty of Applied Sciences University of British Columbia Vancouver, BC Canada

**Keywords:** patient readmission, health care economics, ensemble, prediction model, classification, linear regression resource intensity value, hospital, health care, principal component analysis, PCA

## Abstract

**Background:**

Unplanned patient readmissions within 30 days of discharge pose a substantial challenge in Canadian health care economics. To address this issue, risk stratification, machine learning, and linear regression paradigms have been proposed as potential predictive solutions. Ensemble machine learning methods, such as stacked ensemble models with boosted tree algorithms, have shown promise for early risk identification in specific patient groups.

**Objective:**

This study aims to implement an ensemble model with submodels for structured data, compare metrics, evaluate the impact of optimized data manipulation with principal component analysis on shorter readmissions, and quantitatively verify the causal relationship between expected length of stay (ELOS) and resource intensity weight (RIW) value for a comprehensive economic perspective.

**Methods:**

This retrospective study used Python 3.9 and streamlined libraries to analyze data obtained from the Discharge Abstract Database covering 2016 to 2021. The study used 2 sub–data sets, clinical and geographical data sets, to predict patient readmission and analyze its economic implications, respectively. A stacking classifier ensemble model was used after principal component analysis to predict patient readmission. Linear regression was performed to determine the relationship between RIW and ELOS.

**Results:**

The ensemble model achieved precision and slightly higher recall (0.49 and 0.68), indicating a higher instance of false positives. The model was able to predict cases better than other models in the literature. Per the ensemble model, readmitted women and men aged 40 to 44 and 35 to 39 years, respectively, were more likely to use resources. The regression tables verified the causality of the model and confirmed the trend that patient readmission is much more costly than continued hospital stay without discharge for both the patient and health care system.

**Conclusions:**

This study validates the use of hybrid ensemble models for predicting economic cost models in health care with the goal of reducing the bureaucratic and utility costs associated with hospital readmissions. The availability of robust and efficient predictive models, as demonstrated in this study, can help hospitals focus more on patient care while maintaining low economic costs. This study predicts the relationship between ELOS and RIW, which can indirectly impact patient outcomes by reducing administrative tasks and physicians’ burden, thereby reducing the cost burdens placed on patients. It is recommended that changes to the general ensemble model and linear regressions be made to analyze new numerical data for predicting hospital costs. Ultimately, the proposed work hopes to emphasize the advantages of implementing hybrid ensemble models in forecasting health care economic cost models, empowering hospitals to prioritize patient care while simultaneously decreasing administrative and bureaucratic expenses.

## Introduction

### Background

An open problem that has arisen in Canadian health care economics is the detrimental cost caused by unplanned patient readmissions in hospitals. North American Hospitals have defined patient readmissions as the admittance of patients within 30 days after discharge [1]. In Canada, 1 in 11 patients experience readmittance, resulting in expenses of >2.3 billion Canadian dollars per year [1,2]. Consequently, this enormous expense exemplifies the bidirectional consequences of patient readmission by placing strain on individualized patient care while creating additional expenses for hospitals [1,2]. Furthermore, the COVID-19 pandemic has exacerbated many inequities that revolved around patient readmission owing to inflation. For example, patients with lower income residing in less wealthy neighborhoods were at a higher risk of being readmitted after treatment [3]. Reducing these high readmission rates would prove useful in improving patient outcomes while alleviating financial concerns, for patients and hospitals alike [4,5].

One of the ways to help reduce patient readmissions is to adopt a preventive approach [6]. Risk stratification provides a standardized criterion for assigning a risk status to patients for direct care and to improve overall health outcomes. Machine learning (ML) paradigms have been used to guide clinicians in their efforts to enhance diagnosis and risk stratification [6,7]. Using ML, clinicians can be guided to make accurate diagnoses, improve patient outcomes, and even identify patients at risk of developing certain conditions that can be translatable to readmission and its economic cost. A study by Baruah [8] adopted a detailed approach by analyzing electronic health records using a word convolutional neural network using a “Bag-of-Words.” Although using discharge summaries can allow for the personalization of patient prediction, a work-around for the number of resources required to train a high-throughput model such as word convolutional neural network is of high concern [8]. Furthermore, Baruah’s [8] model was limited in addressing the high class imbalance in shorter time frame readmission tasks in contrast to longer time frame readmission tasks [8]. Solving this short time frame readmission problem can allow for a faster prevention of unplanned patient readmission [8,9].

Although deep learning models were used for risk stratification in health care, they had limited success because of the large amount of data required for training [7,8]. In addition, incorporating comorbidities and their time periods in models could lead to the confounding of other variables [9-12]. However, Ben-Assuli et al [13] found that using multiple time periods and ensemble ML methods on large-scale data enabled early risk identification in specific patient groups [13]. Stacked ensemble models, including those with boosted tree algorithms, demonstrated strong performance in predicting unplanned patient readmissions by reducing bias from individual models and sensitivities to rare classes [9,10]. These models also offered better interpretability for health care workers and nonexperts in ML, thanks to their transparent results [9-11].

After determining whether the patients will be readmitted within the next few days, the economic consequences to both the hospital and the patient will be estimated [14,15]. This involves finding the causal relationship between patients’ expected length of stay (ELOS) and their resource use, which are both continuous variables for determining the economic aftermath of hospital readmission [14,15]. However, if given a time period, linear regressions may prove useful in predicting and comparing the trends behind the relationships between variables such as ELOS and readmission in real time [14,15].

### Goal of This Study

The objectives of the proposed work were 3-fold. The first, main goal of the project was to implement an ensemble model with individual submodels on the structured data and compare the resulting metrics to metrics resulting from other models that have also explored patient readmission in a heart-disease context. The second goal was to determine the contribution of optimized data manipulation through principal component analysis (PCA) to solving the problem of shorter time frame readmissions. The study also aimed to verify the causal relationship between the ELOS and resource intensity weight (RIW) value. Providing an understanding of this relationship in a quantitative and causal manner can allow for an in-depth economic perspective, as opposed to only readmittance within 30 days.

Ultimately, the economic and predictive aspects of this model are intended to provide a view on resource allocation for health institutes to better predict readmittance and improve patient-clinician outcomes [5].

## Methods

### Resources Used

#### Population Study

**Figure 1:**
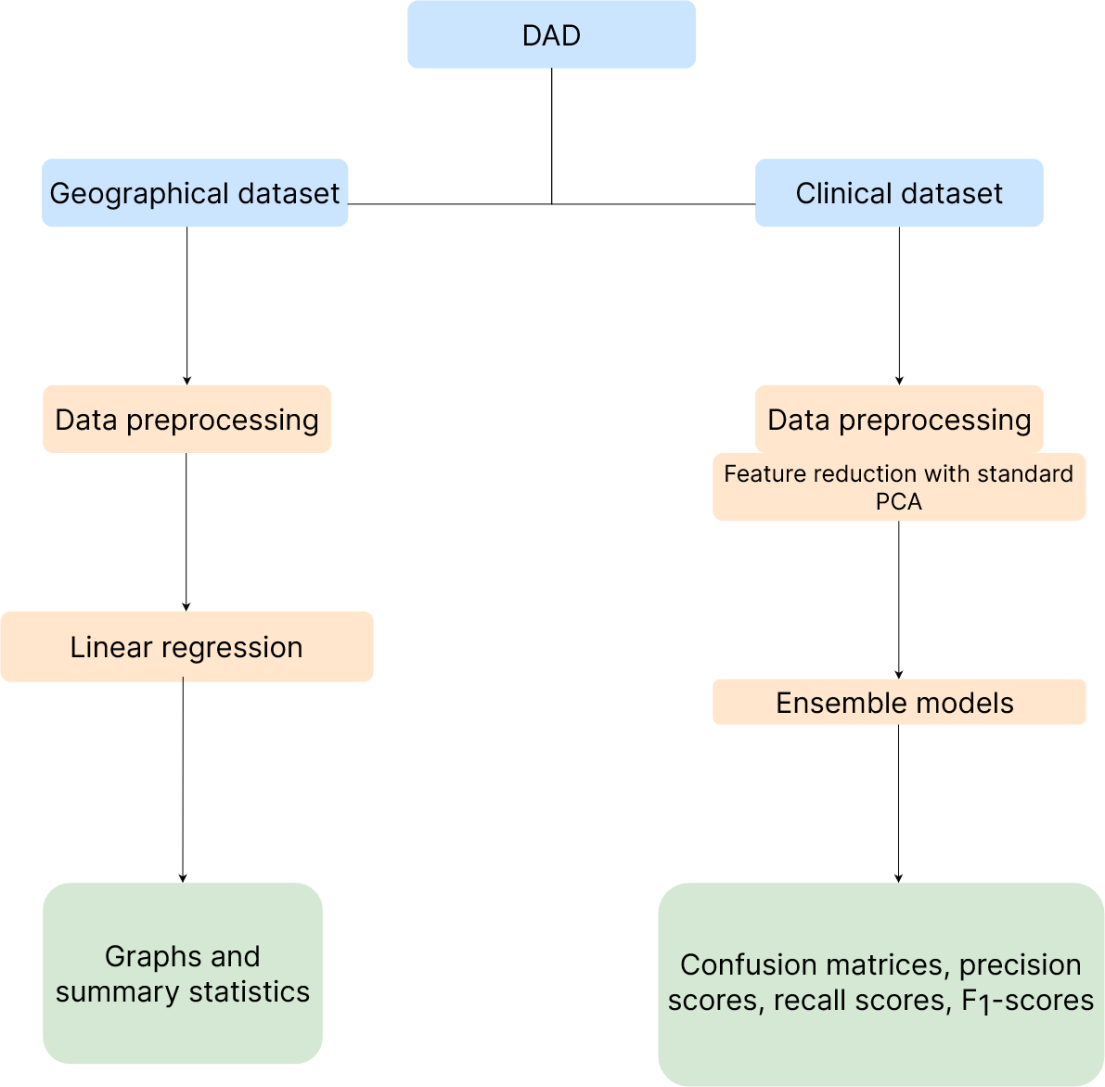
Study workflow: data collection (blue), data preparation and machine learning implementation (orange), and outputs (green). DAD: Discharge Abstract Database; PCA: principal component analysis.

The study used a systematic methodology with Python 3.9 and streamlined libraries to analyze the data obtained from the Discharge Abstract Database (DAD) covering 2016 to 2021 [16]. Access to the database was facilitated through the Abacus Data Network, a collaborative effort between several universities [17]. The study used 2 sub–data sets, clinical and geographical data sets, to predict patient readmission and analyze economic implications, respectively. The comprehensive documentation provided by Statistics Canada allowed for a robust analysis of the data set. The workflow, illustrated in Figure 1, shows the process of data analysis and visualization using the matplotlib and seaborn libraries.

#### Design

Similar to the study by Baruah [8], during clinical and geographical preprocessing, individuals were screened for specific criteria. Using the International Classification of Diseases, 10th revision (ICD-10) and major complication or comorbidity (MCC) codes similar to the models by Baruah [8] and Liu et al [18], the examination of adult patients and exclusion of individuals aged <18 years were performed to prevent any confounding variables “spilling” onto both models. The ICD-10 PCA codes for the diseases included I092, I098, I099, I100, I101, I11, I13, I500, I501, I509, I516, I518, I519, I520, I521, and I528 [16,17]. As for MCC codes, only code 5 corresponded to cardiovascular diseases [16,17]. Factors that were not considered were clinical gestation of delivery (“GES_AGRP”) along with weight group (“WGT_GRP”), as they were only a direct consequence of the age group that was eliminated [16,17].

#### Clinical Data Set

##### Clinical Preprocessing

**Figure 2:**
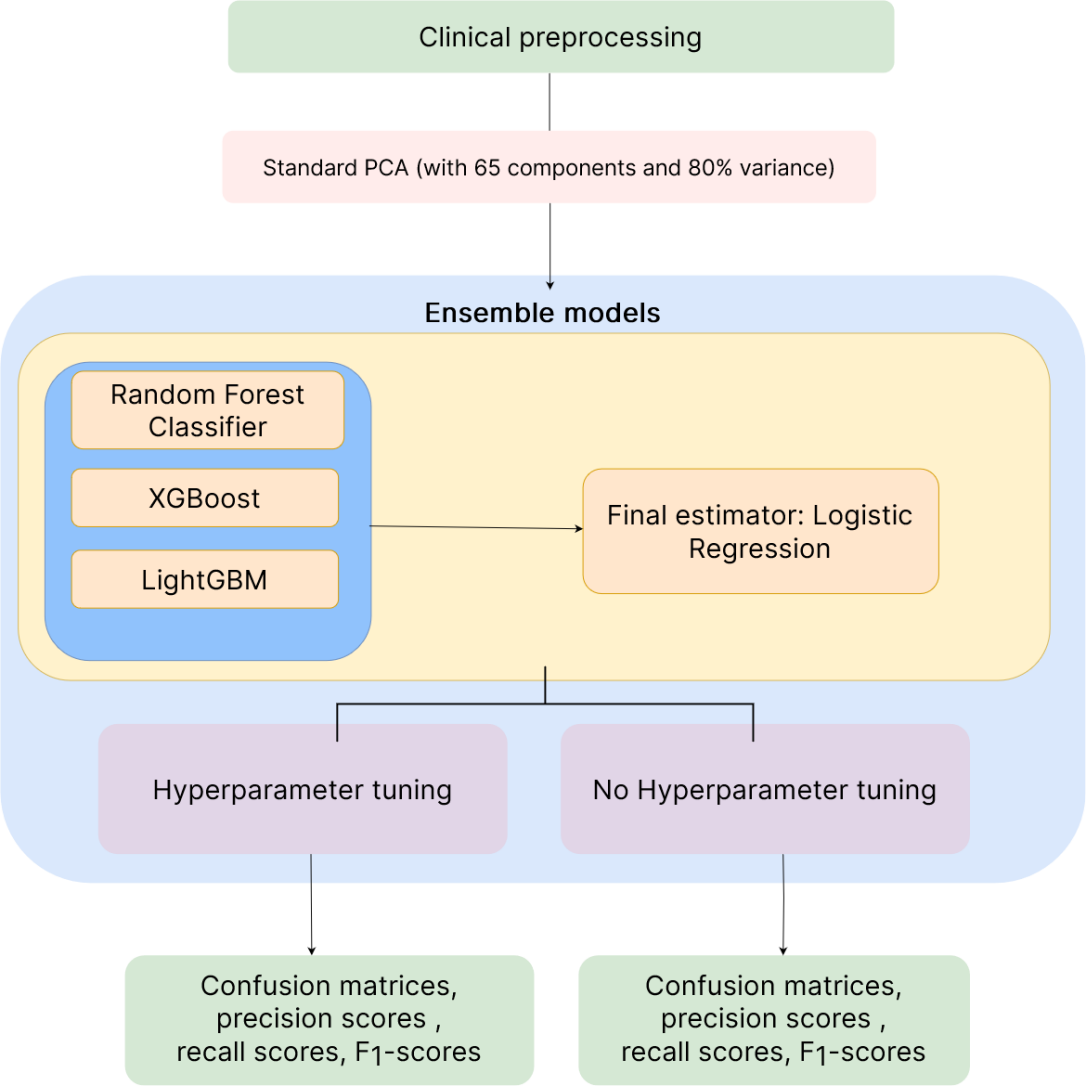
Clinical workflow: data collection (blue), data preparation and machine learning implementation (orange), implementations (purple), and outputs (green). LGBM: LightGBM; PCA: principal component analysis.

Figure 2 shows the manipulation done and models trained on the clinical data set of DAD. Isolating for a group of patients who share similar clinical characteristics or medical conditions can be useful for identifying trends and patterns in patient care and outcomes, as well as for conducting research on specific medical conditions such as heart disease.

A clinical preprocessing step was performed to isolate for specific criteria and remove any potential confounding variables. Arbitrary admission and discharge dates were chosen based on previous calculations to avoid errors or inconsistencies in the data set. To ensure that the minimum number of relative admission dates was ≥0, dates were shifted to a minimum of January 5 of the corresponding data set year. This adjustment enabled the creation of the “LTORET30Days” columns. For feature selection and dimensionality reduction, PCA was used, as it was a common methodology used for high-dimensionality data sets.

##### PCA Process

According to the PCA criterion, the components to use were described by the minimum number of features required to obtain a cumulative variance of at least 80% [19,20]. The aim was to reduce the dimensionality of the feature space while retaining as much of the original variance as possible [19-21]. After obtaining an encoded vector in the form of an array, the data were run through several ensemble algorithms. The ensemble algorithm consisted of several submodels, including random forest classifiers, XGBoost (XGB), and LightGBM (LGBM). Each subclassifier’s output was stacked, allowing for a logistic regression to learn the weighted distribution of the subclassifiers to ensure high predictive accuracy. After dimensionality reduction and splitting into training and testing data sets, the final sample size was n=83,083 for nonreadmitted patients and n=10,271 for readmitted patients.

##### Submodels: LGBM and XGB

LGBM and XGB presented a relative advantage with regard to efficient computation and high accuracy on a wide range of data sets, including those with high dimensionality and categorical features [22-25]. Both methods required sequential decision tree generation via error combination or level-wise tree growth. Having dimensionality reduced data would have decreased the maximum function, *δ_loss_*, for LGBM, allowing for lower error and lower changes in *∇_prediction_* [26,27]. Similarly, it was extrapolated that a higher maximum depth for XGB would be achieved, as the number of features was lower [26,27]. An in-depth analysis about LGBM and XGB can be found in Figures S1-S11 in Multimedia Appendix 1.

##### Random Forest

Random forest was chosen to improve the interpretability of the model when used in conjunction with PCA [21]. As the data set had a large number of features, random forest’s computational cost was high. However, after performing dimensionality reduction using PCA, the computational cost of random forest was substantially reduced, making it a practical option for large data sets [21]. During the testing phase, the random forest classifier predicted the final decision of a new data point, noted by *C^B^rf^(x)^*, by aggregating the prediction results of all decision trees using a majority vote. The classifier selected the class with the highest number of votes as the final prediction, resulting in an accurate and interpretable model. The algorithm design for random forest is formulated in Multimedia Appendix 1.

##### Ensemble Models: Logistic Regression

The ensemble model used in this study was a stacking classifier model with a metamodel (final estimator), which was a logistic regression model [26]. The metamodel took the outputs of the base models as inputs and optimally combined their predictions to ensure high predictive performance [26]. The ensemble model consisted of 4 base models and was defined, trained, and tested using the scikit-learn's ensemble module, which was the default. This produced an optimal workflow, which is presented in Figure 2. Detailed formalisms are provided in Multimedia Appendix 1.

##### Hyperparameter Tuning

To optimize the performance of each base model, hyperparameter tuning was done using a range of values for each parameter [27]. The models were evaluated based on their *F*_1_-score or recall, and scikit-learn's GridSearchCV and RandomizedSearchCV were used to fine-tune the parameters [27].

In addition, a custom function was used to optimize the final estimator of the stacking model, specifically for the logistic regression component [21]. Table 1 lists the parameters used for all the models. By tuning the hyperparameters of the base models and customizing the final estimator for the stacking model, we aimed to improve the overall performance and accuracy of the ML model [27-30].

**Table 1:** Tuned parameters organized according to submodels and estimators.

Model	Parameters
XGB^a^	max_depth, n_estimators, and learning_rate
Random forest	bootstrap and max_depth
LGBM^b^	learning_rate, n_estimators, num_leaves, min_child_samples, subsample, max_depth, colsample_bytree, reg_alpha, reg_lambda, and min_data_in_leaf
Logistic regression (stacking ensemble)	solver, penalty, and C

^a^XGB: XGBoost.

^b^LGBM: LightGBM.

### Evaluation of the Ensemble Model Outcomes

#### Evaluation Metrics

Statistical analysis was performed to ensure that the model was robust in and valid for improving the patient outcomes. Three evaluation metrics were used to evaluate the robustness of the model.

1. Precision is the ratio between the true positive observations and total positive observations obtained from the confusion matrix [31]. In other words, it provides the number of retrieved items that are relevant. This was a crucial quantity, especially given that there was high class imbalance:

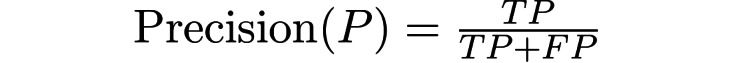

2. Recall is the ratio between the number of true positives and the sum of the number of true positives and number of false negatives [31]. The recall score provides the number of relevant items retrieved [31]. The recall score was useful in determining the model validity regardless of class imbalance owing to the measurement of false negatives:

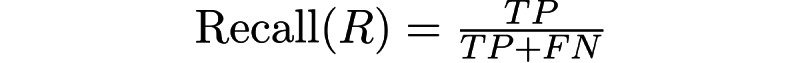

3. Balancing the 2 quantities required the use of *F*_1_-score, which serves as the harmonic mean of the precision score and recall score [31]:

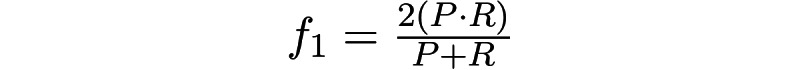

All the scores for the hyperparameter-tuned data were plotted on a bar graph to ensure a clear presentation of the data [31].

#### Geographical Data Set

##### Feature Selections

To determine the relationship between ELOS and RIW, 2 continuous variables that have been shown to be positively correlated with improved patient outcomes, a linear regression analysis was conducted [32-34]. RIW is a weighted measure of the anticipated use of resources associated with various demographic, diagnostic, and surgical procedure characteristics of an individual [29,30]. Multimedia Appendix 1 discusses the requirements for the calculation and formulation of RIW [35]. Therefore, linear regression analysis has the potential to provide a quantifiable measure of the correlation between these variables, thereby meeting the third objective of the research paper, which is to conduct an in-depth economic analysis [29].

**Figure 3:**
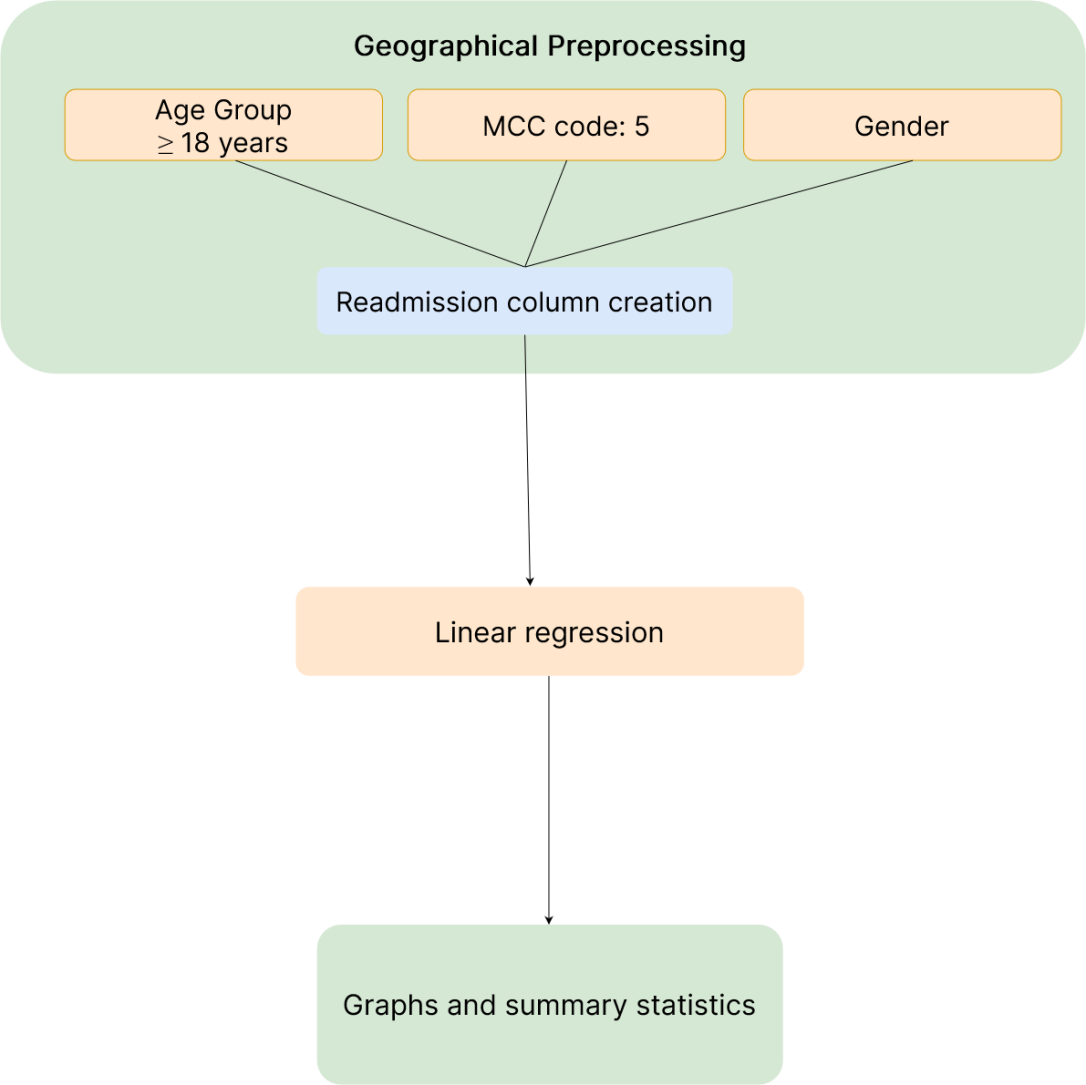
Geographic workflow: data collection (blue), data preparation and regression implementation (orange), and outputs (green). MCC: major complication or comorbidity.

To ensure that the results were not biased by confounding factors, the linear regression analyses were conducted separately for each age group, gender, and readmission column class [33]. This approach ensured that any potential effects of these variables were taken into account. Figure 3 demonstrates the approach used for the geographical data sets.

##### Main and Controlled Geographic Data Set Variables

After the data were isolated for individuals aged >18 years and the MCC codes, Python pandas were used to condition the data set onto covariates. The entire data set was then placed into specific clusters based on this condition. First, individuals were clustered according to whether they had the same patient readmission column value, and then they were split by gender. Afterward, each data point was separated into age clusters. There were 2 gender data clusters for each of the 2 readmitted clusters and 15 age clusters for each of the 4 resulting clusters, resulting in 60 linear regressions being performed. To clarify, the main independent variable was ELOS, and the dependent variable was RIW. The data were split to verify the hypothesis that there was indeed an economic benefit to extending a patient’s length of stay rather than being readmitted.

### Ethical Considerations

This study was exempt from research ethics review, as it was a secondary analysis of research data. As data were received directly from acute care facilities or from their respective health or regional authority or ministry or department of health, facilities in all provinces and territories except Quebec were required to report. The authors do not claim any right to the data, as they are the property of Statistics Canada along with the Abacus Student Network [16,17].

## Results

The results of the main study are presented in this section. The results for the PCA, feature selection stages, and more data can be found in section B in Multimedia Appendix 1.

### Classification Reports

The evaluation metrics for the ensemble model were presented using classification reports (Table 2). In this context, class 0 represented the model’s performance for the negative class (ie, patients who did not return within 30 days), and class 1 represented the model’s performance for the positive class (ie, patients who did return within 30 days). The support column indicated how many examples of each class were there in the test set.

**Table 2:** Classification reports for different models.^a^

Model type and class		Precision	Recall	*F*_1_-score
**XGBoost**				
	0^b^	0.92	0.99	0.95
	1^c^	0.79	0.31	0.44
**Random forest**				
	0	0.93	0.97	0.95
	1	0.65	0.39	0.48
**LightGBM**				
	0	0.96	0.91	0.93
	1	0.49	0.68	0.57
**Ensemble model^d^**				
	0	0.96	0.91	0.93
	1	0.49	0.68	0.57

^a^All of these models have been hyperparameter tuned.

^b^For all models, class 0 contains n=16,592.

^c^For all models, class 1 contains n=2079.

^d^Tuned submodels and tuned ensemble models.

### Correlation Between Inpatient RIW and ELOS

A least squares linear regression model was fitted to the ELOS and RIW value columns of a geographical data set, and a summary of the best-fitted lines was obtained (Tables 3-5). The corresponding plots (Figures S6 and S7 in Multimedia Appendix 1) and tables (Tables 6-8) produced by the least squares linear regression was also obtained, and the data were stratified by readmission status, age group, and gender. The coefficient of determination (*R*^2^) was included, and it took a value between 0 and 1, providing a sense of how correlated the 2 variables were, with a value of 1 indicating perfect correlation. Note that all age groups had a *P*<.001. The threshold was chosen as it was expected highly correlated and the root mean square error was used to measure the distance between predicted and actual values.

**Table 3:** Regression lines fitted for women who were readmitted within 30 days, separated by age groups.

Age group (years)	Slope (expected length of stay)	Intercept	*R*^2^ adjusted	RMSE^a^	*F* statistics	Sample size, n
18-24	0.196661	–0.038496	0.469363	1.222708	67.339641	76
25-29	0.236855	–0.032451	0.482341	2.067290	128.653235	138
30-34	0.195582	0.009696	0.308630	2.507248	69.299619	154
35-39	0.455326	–1.431780	0.587717	3.356237	213.402169	150
40-44	0.776407	–3.014587	0.573208	6.474491	284.385704	212
45-49	0.355912	–0.681188	0.626838	2.286141	572.132763	341
50-54	0.338375	–0.350636	0.656287	1.246735	990.071951	519
55-59	0.269042	0.021663	0.500513	1.631754	776.589838	775
60-64	0.266451	0.023355	0.401983	1.507652	755.872164	1124
65-69	0.361558	–0.433117	0.562777	1.813054	1821.045541	1415
70-74	0.346215	–0.373987	0.455203	2.111974	1393.857933	1668
75-79	0.280143	–0.088512	0.499735	1.388378	1795.095118	1797
>80	0.280403	–0.140523	0.321200	1.600127	1975.140727	4173

^a^RMSE: root mean squared error.

**Table 4:** The 4 types of submodels.

Ensemble model	Tuned submodels (Y^a^ or N^b^)	Tuned LR^c^ (Y or N)
1	N	N
2	N	Y
3	Y	N
4	Y	Y

^a^Y: yes.

^b^N: no.

^c^LR: logistic regression.

**Table 5:** Comparison of existing literature values.

Author name or literature values	Description of model	Comparison to current literature values with precision, recall, and *F*_1_-score
Sharma et al [36]: “Predicting 30-Day Readmissions in Patients With Heart Failure Using Administrative Data: A Machine Learning Approach”	Sharma et al’s [36] implementation of XGBoost created a precision-recall curve. Their precision and recall balance for class 1 was significantly lower than that of the ensemble model. However, the ensemble model proposed in this work allows for high balance.	Sharma et al [36] used a precision-recall curve to evaluate the performance of their model. The bias-variance trade-off was observed to be high during the analysis. The primary evaluation metric used in the study was the AUC^a^, which was not used for the proposed work here.
Jamei et al [37]: “Predicting All-Cause Risk of 30 Day Hospital Readmissions Using Artificial Neural Networks (ANN)”	Jamei et al [37] predicted patient readmission using a neural network. Their precision and recall balance was skewed, as the precision for their models was low, yet the recall was high. This results in high variance but low bias.	The following scores were given for the 2-layer neural network of Jamei et al [37], with the number of features being high: precision=23%, recall=59%, and *F*_1_-score=16.5%. This indicates that the proposed model in this work has a significant advantage compared with an ANN^b^.
Ho et al [38]: “Predicting Readmission at Early Hospitalization Using Electronic Health Data: A Customized Model Development”	Ho et al [38] predicted a within a 24 month period. The model they used was an XGBoost Model having access to specific laboratory data in addition to the variables addressed in our work.	The following scores were present in the readmission stage: recall score of 80% and a precision score of 76%. Although these scores may be higher overall due to the presence of more personalized data such as specific laboratory results for each patient. Furthermore, Ho et al [38], does not seem to stratify based on specific diseases which could result in bias effecting this score.

^a^AUC: area under the curve.

^b^ANN: artificial neural networks.

**Table 6:** Regression lines fitted for men who were not readmitted within 30 days, separated by age groups.

Age group (years)	Slope (expected length of stay)	Intercept	*R*^2^ adjusted	RMSE^a^	*F* statistics	Sample size, n
18-24	0.133004	0.603021	0.301397	1.165577	228.362523	528
25-29	0.332606	–0.276038	0.614197	1.609255	718.990166	452
30-34	0.492425	–1.069907	0.697588	2.279228	1521.145371	660
35-39	0.447525	–0.844292	0.653325	2.407347	1902.504340	1010
40-44	0.466519	–0.840117	0.647550	2.075903	3118.868054	1698
45-49	0.380460	–0.308439	0.583264	1.770296	3957.666828	2828
50-54	0.420954	–0.557944	0.626193	2.074092	7922.913006	4730
55-59	0.410799	–0.440937	0.570193	2.223704	9108.272364	6866
60-64	0.421378	–0.502756	0.471093	2.769979	7587.014726	8518
65-69	0.341375	–0.053999	0.514460	2.144153	10,030.832915	9467
70-74	0.327909	–0.015719	0.476918	1.992737	8977.140732	9846
75-79	0.331579	–0.114296	0.447201	2.177262	7031.801936	8692
>80	0.296201	–0.082061	0.269552	2.414906	6006.483269	16,275

^a^RMSE: root mean squared error.

**Table 7:** Regression lines fitted for men who were readmitted within 30 days, separated by age groups.

Age group (years)	Slope (expected length of stay)	Intercept	*R*^2^ adjusted	RMSE^a^	*F* statistics	Sample size, n
18-24	0.13304	–0.123251	0.659408	1.936411	159.756983	83
25-29	0.434780	–1.444296	0.563547	3.009955	123.663857	96
30-34	0.205049	0.338259	0.414037	1.963718	109.108782	154
35-39	0.503076	–1.434408	0.597649	3.516315	387.201489	261
40-44	0.319638	–0.426751	0.633530	1.556234	708.053550	410
45-49	0.321520	–0.251261	0.543536	2.238206	883.346712	742
50-54	0.305131	–0.157782	0.546973	1.329511	1546.437115	1281
55-59	0.336327	–0.291609	0.516734	1.844321	1963.077772	1836
60-64	0.387830	–0.541003	0.524773	1.978364	2742.875413	2484
65-69	0.356209	–0.334169	0.526643	1.919789	3103.957220	2790
70-74	0.315150	–0.190539	0.503276	1.686930	2989.908128	2951
75-79	0.330981	–0.242378	0.515653	1.935420	2699.848355	2536
>80	0.320212	–0.272116	0.388353	1.864184	2708.979941	4266

^a^RMSE: root mean squared error.

**Table 8:** Regression lines fitted for women who were not readmitted within 30 days, separated by age groups.

Age group (years)	Slope (expected length of stay)	Intercept	*R*^2^ adjusted	RMSE^a^	*F* statistics	Sample size, n
18-24	0.290987	–0.168320	0.650458	1.837020	733.188372	306
25-29	0.340779	–0.378737	0.570807	2.726389	589.170872	445
30-34	0.324827	–0.284349	0.584901	2.048568	789.076632	562
35-39	0.368889	–0.515399	0.631981	1.725110	1102.474089	644
40-44	0.253023	0.147074	0.531883	1.569319	1070.319261	944
45-49	0.324630	–0.232875	0.627709	1.493320	2394.218647	1422
50-54	0.301186	–0.073365	0.478817	1.655429	2019.326558	2200
55-59	0.389959	–0.484006	0.576303	1.886088	4468.179114	3287
60-64	0.339517	–0.190579	0.422514	2.111166	3013.638447	4121
65-69	0.297055	–0.029449	0.504601	1.616991	5405.580063	5309
70-74	0.333896	–0.262753	0.482080	2.016495	5622.958698	6043
75-79	0.348289	–0.358721	0.439302	2.057815	5139.697471	6562
>80	0.266803	–0.034627	0.179332	2.489782	4115.368718	18,835

^a^RMSE: root mean squared error.

## Discussion

The proposed work aimed to use ensemble models and linear regressions for predicting patient readmissions and analyzing their economic consequences [32,33]. The results of this study demonstrate the potential of these models to accurately predict readmissions with a balanced degree of recall and precision, which could help health care providers identify patients who are at risk of readmission and take proactive measures to prevent it.

### Notes About the Study

Although the study used cutting-edge algorithms for classification and regression, there are several critical notes that must be considered [39]. The primary evaluation metrics for the models were recall and *F*_1_-scores, with a slight preference for false positives over false negatives to decrease the likelihood of unplanned readmissions [40]. However, it is crucial to note that this approach may not be suitable for all health care scenarios and should be evaluated on a case-by-case basis [41].

Another crucial consideration is the computational cost associated with clinical and graphical data [41]. Although the analysis for this study only took 2 to 3 hours, it is essential to consider the computational requirements for more substantial studies, particularly those with larger data sets or more complex models [42]. The computational cost may impact the feasibility of the study, and efficient models may be necessary to ensure valid and reliable results [42].

In addition, some features in the geographical data, such as the case mix group diagnosis type, could not be split in the geographical data sets because of their high computational cost. This could lead to omitted variable bias and negatively affect the models’ accuracy [43]. As the impact of not splitting these features was not taken into account in this study, future research should carefully evaluate the potential impact of not splitting features and consider alternatives to reduce the computational cost [43].

### Clinical Data Set Result Analysis

In this section, the clinical data set results are analyzed and compared with those of other existing models in the literature.

#### The Effect of PCA on the Study and the Bias-Variance Trade-off

The use of PCA offered several advantages. The selection of the components that describe the minimum number of features required to achieve a cumulative variance of at least 80% proved to be effective in preventing overfitting [31,44-46]. The data set had high dimensionality and a substantial number of data points, which would have led to high bias and low variance without the use of PCA [39]. This, in turn, would have resulted in a lower precision rate than recall rate. However, PCA prevented this issue by reducing the number of features in the model and substantially increasing computational efficiency [47].

Moreover, PCA eliminated the potential for collinearity, which can create unstable and unreliable estimates of the model parameters [39]. Collinearity makes it difficult to determine the unique contribution of each variable to the outcome [41]. Upon computing the covariance matrix and performing an eigenvector decomposition, the resulting eigenvectors were orthogonal to each other, thereby eliminating the presence of collinearity.

Furthermore, the implementation of PCA in conjunction with stacked classifiers enabled a higher interpretability of the models [42]. Stacked models can be challenging to interpret in high-dimensional data, as the layers can contribute to a high level of complexity [43]. Moreover, the curse of dimensionality and collinearity can make it difficult for models to isolate specific features, thereby decreasing transparency [43]. However, the addition of PCA allowed for a more comprehensive and explained model, as reflected in the submodel and ensemble model analyses in the subsequent sections.

#### Submodel Analyses

This study found that although the hyperparameter-tuned XGB model outperformed its base model, it was still less accurate than the other individual submodels. This result is consistent with a previous study conducted in Alberta that also found that XGB models did not provide substantial information on patient readmissions [36]. However, the tuned XGB model performed better than its base model and had a higher precision and recall score, indicating a better balance between precision and recall for both classes relative to the default XGB model.

By contrast, both the tuned random forest and LGBM models (Tables 6 and 7, respectively) demonstrated superior performance compared with their base models (Table S1 in Multimedia Appendix 1) in predicting patient readmission for class 1, as evidenced by their higher *F*_1_-score and precision. The recall for class 1 was lower for the tuned random forest model, whereas it was higher for the tuned LGBM model. LGBM was shown to balance a slightly higher recall rate and precision rate than its other decision tree counterparts, allowing it to provide substantial information regarding the use of this model.

#### Final Estimator Analysis

The ensemble model was created to ensure minimization and offset bias and variance between each of the models in discussion [44,45]. The 4 types of ensemble models and their classification reports are listed in Table 4 and Table S2 in Multimedia Appendix 1, respectively.

Upon analyzing the data, it was observed that the default model configuration, which consisted of default submodels and a default final estimator logistic regression, exhibited high precision (0.92) and recall (0.98) for nonreadmitted patients (class 0). However, its ability to predict readmissions (class 1) was comparatively weaker, as evidenced by the lower *F*_1_-score (0.46), precision (0.69), and recall (0.35) for class 1.

The second configuration, which used default submodels with a tuned logistic regression final estimator, demonstrated an improvement in the *F*_1_-score (0.56) for class 1. Nonetheless, its precision (0.47) and recall (0.68) for class 1 remained lower than those for class 0.

The third configuration, which used tuned submodels with a default final estimator logistic regression, yielded high precision (0.92) and recall (0.99) for class 0. However, its performance in predicting readmissions (class 1) was weaker, with a precision of 0.77 and recall of 0.30, leading to an *F*_1_-score of 0.43.

The fourth configuration, in which both submodels and final estimator logistic regression were tuned, resulted in the highest *F*_1_-score (0.57) for class 1, indicating a better performance in predicting patient readmissions. Nevertheless, its precision (0.49) and recall (0.68) for class 1 remained lower than those for class 0.

The overall tuned ensemble model, when compared with the submodels, is identical to the LGBM model, as although recall is favored, the balance between precision and recall for class 1, compared with the other models, is useful in preventing too many false positives from occurring.

#### Comparison of Tuned Ensemble Models With Literature Value Predictions

The results of this study are not comparable with Baruah’s [8] values because of the presence of unstructured data types, which cannot serve as a useful comparison to ordered data sets such as the DAD. However, other studies have used the DAD or other similar structured data before. The existing literature review comparisons with 30-day short-term studies are presented in Table 5.

Note that this list is not exhaustive and that there may be other studies that potentially use stacking classifier models and show better results. The comparison with other studies shows that the model has the potential to be viable and robust, but more tuning and comparison between submodels need to be performed.

#### Limitations of the Clinical Data Set Analysis

One notable limitation of the clinical data set used in this study was the high class imbalance problem. Specifically, there were considerably more training points for class 0 than for class 1, with n=83,083 for class 0 and n=10,271 for class 1. This issue could have led to the trained model being more prone to producing false negatives than to producing false positives, as it was more familiar with class 0 instances and thus had a tendency to classify more instances as class 0 [48,49]. Consequently, this limitation could have negatively impacted the overall performance and accuracy of the model, as well as the reliability of the predictions it produced [50].

Another limitation of the data set was the encoding of the data, which could have influenced the interpretability and accuracy of the model. Specifically, if the model interpreted the encoded data as ordinal, it could have altered the ordinality of the classifier, thereby influencing the classification results. This limitation could have impacted the ability of the model to identify the most relevant features for predicting patient readmission, reducing its interpretability [49]. Moreover, this limitation could have adversely impacted the accuracy of the model, as the model may have learned from the encoded data instead of the underlying features, resulting in a less accurate prediction of patient readmission [50].

Finally, the data set’s lack of information about the specific principal component that contributed to the accurate prediction of the patient data set was another limitation. This limitation could have constrained the model’s ability to explain how the variables were associated with patient readmission, resulting in a lack of transparency in the model’s predictions and reduced ability to elucidate the rationale behind its decision-making process. As such, identifying the principal components that contribute to the accurate prediction of the patient data set is critical to improving the interpretability and reliability of the model.

### Geographical Data Set Result Analysis

#### Causality of the Linear Regression Model

The study results suggested that the model could potentially establish a causal relationship (albeit with a proper regression type) between ELOS and RIW. The anticipated hypothesis was well supported by the tables presented earlier, indicating the importance of the model. The analysis involved an explicit model of a continuous outcome (RIW) that was affected by a measured continuous variable (ELOS), and the results showed a notable impact. This finding encourages the establishment of causality in the relationship between ELOS and RIW.

#### ELOS Effects on RIW and Fit of the Linear Regression

The relationship between ELOS and RIW was investigated through a linear regression analysis, which produced the coefficient (slope) from the ELOS variables. The study findings indicated that more resources were expended and more time was spent among women aged 40 to 44 years who were readmitted than among those who were not readmitted. In addition, more resources were expended for men aged 35 to 39 years (Table 7) who were readmitted than for their nonreadmitted counterparts. Surprisingly, most of the slopes associated with ELOS are uniform in nature and are approximately the same across ages. However, a comparison between the results also suggested that ELOS had a significant effect on RIW owing to the low *P* values.

The *F* test of overall significance was used to ascertain that the model was better suited than a model with no independent variables [51,52]. All the models had *F* statistic values significantly greater than their critical *F* values, which suggested that the linear regression model was a relatively accurate estimate of the relationship between ELOS and RIW.

However, the root mean squared error and *R*^2^ values suggested otherwise. There was a high degree of error compared with the slope. The low *R*^2^ values across all the studies implied that linear regression was not a good fit, which could imply that further data clustering into groups was necessary or that further manipulation of the data to perform a different regression was needed. These results were reasonable, considering that the function was not 1-to-1, as demonstrated by the graphs in Figures S6 and S7 in Multimedia Appendix 1.

### Future Directions

Many fundamental aspects of both the ensemble model and linear regression remain unexplored.

Therefore, the suggested future implementations for the ensemble model are as follows:

- Including unstructured data (such as clinical data and text notes) in analysis by a deep neural network and performing logistic regression on all the models to give individuality to a specific patient [18].
- Using deep learning neural networks as a final estimator for the ensemble model and outputting evaluation metrics [53].
- Adding more submodels and optimizing for computational resources such as space, time, and memory [53].

The suggested improvements for the linear regression include are as follows:

- An instrumental variable that measures the relationship between ELOS and a selection decision variable should be implemented. The instrumental variables should only be involved in the selection decision process. Afterward, the relationship between RIW and the selection decision variable should be measured to ensure low omitted variable biases [54].
- Logistic regression (logistic by the coefficients) should be performed to ensure that root mean squared error is minimized and a more accurate relationship between the ELOS and RIW can be derived [55].

These applications can allow for a more in-depth analysis and provide a multifaceted perspective in the fields of ML, econometrics, and health care interventions.

### Conclusions

The study’s implications are to validate the use of hybrid ensemble models and attempt to predict economic cost prediction models. The availability of robust and efficient predictive models, such as the one presented in this study, can enable hospitals to focus more on patients and less on the utility and bureaucratic costs associated with their readmission. As demonstrated by the evaluation metrics, the ensemble model plays a critical role in ensuring more precise results overall. By implementing a crowdsourcing approach, the model can also estimate the resources required to control future epidemics in an easier, time-sensitive manner while maintaining low economic costs. This is particularly relevant in decentralized, universal, publicly funded countries such as Canada, where high inflation on medical equipment, technologies, and maintenance has been observed in the aftermath of the COVID-19 pandemic.

Predicting the relationship between ELOS and RIW can also indirectly predict patient outcomes by reducing bureaucratic and utility costs, thereby reducing the cost burden placed on patients to implement administrative tasks and on physicians to ensure their execution. The ensemble model also considers the specific disease type, and the encoding process has resulted in the classification data being ordinal in nature, which takes into account patient utility in addition to risk stratification.

The linear regression has considered the differences in continuous variables while also allowing for a clear difference in the clustered groups. Further exploration of the cost-benefit economic model can enable hospitals to ensure more cost-free, patient-friendly outcomes. It is recommended that after making several changes to the general ensemble model and the linear regressions, they be used to analyze new and incoming numerical hospital cost data.

## Appendix

Multimedia Appendix 1 In-depth descriptions and mathematical formalisms for all of the submodels and ensemble models, cumulative variance and principal component analysis results and confusion matrices of all of the previous models and graphs for the linear regression, all the codes, and references.

## Abbreviations

DAD: Discharge Abstract Database
ELOS: expected length of stay
ICD-10: International Classification of Diseases, 10th revision
LGBM: LightGBM
MCC: major complication or comorbidity
ML: machine learning
PCA: principal component analysis
RIW: resource intensity weight
XGB: XGBoost

## Data Availability

All codes have been made available by the authors in Multimedia Appendix 1. The data set used in this study was obtained from Statistics Canada and is subject to copyright owned by Statistics Canada.
