# Supplementary material for: Machine Learning and Causal Approaches to Predict Readmissions and Its Economic Consequences Among Canadian Patients With Heart Disease: Retrospective Study
Source: JMIR Form Res. 2023 May 26;7:e41725. doi: 10.2196/41725 (PMC10257109; doi:10.2196/41725)
Supplement: Multimedia Appendix 1 [file formative_v7i1e41725_app1.docx]

# Table Of Contents

[**Appendix A: Model Formalisms 2**](#_slijz0p4i0fc)

[**Appendix B: Results 4**](#_d6238srft70z)

[Cumulative Variance For PCA (n = 65 components) 4](#_uucicke9guih)

[Confusion Matrices 5](#_dkx514udbhf7)

[Classification reports 8](#_rfbe54x152wc)

[**Appendix C: Code 14**](#_jw5yhvtxltj5)

[Clinical Dataset Notebook 14](#_gkxbh6chrqm8)

[Geo Dataset Notebook 30](#_2g3a29acus72)

#

# Appendix A: Model Formalisms

**LightGBM and XGBoost :**

Both LightGBM and XGBoost are scalable, distributed gradient-boosted discussion tree models which have seen a rise in popularity in recent years . In both methods, a sequential decision tree will be built by another tree. The difference between XGBoost and LightGBM is level-wise tree growth and leaf-wise strategy [1]. For LightGBM, each tree will be built using an error based combination. Tree 1 would have a small [
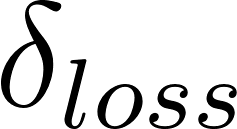
](https://www.codecogs.com/eqnedit.php?latex=%20%5Cdelta%20_%7Bloss%7D%20#0) function than each of its descendants Tree 2 and Tree 3. The higher the maximum [
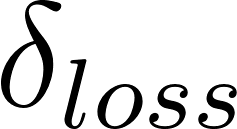
](https://www.codecogs.com/eqnedit.php?latex=%20%5Cdelta%20_%7Bloss%7D%20#0) function, the higher the error [1]. The “growing” process stops when a similar maximum [
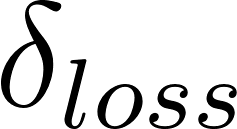
](https://www.codecogs.com/eqnedit.php?latex=%20%5Cdelta%20_%7Bloss%7D%20#0) function occurs. Predictions will then be made by the sum of the error for all of these trees (MAE). Hence, the data is split into nodes with the highest loss change. Mathematically, the maximum [
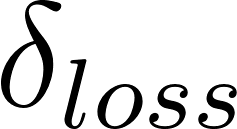
](https://www.codecogs.com/eqnedit.php?latex=%20%5Cdelta%20_%7Bloss%7D%20#0) or [
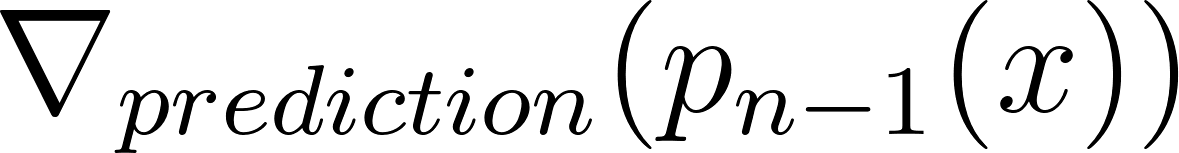
](https://www.codecogs.com/eqnedit.php?latex=%20%5Cnabla_%7Bprediction%7D%20(p_%7Bn-1%7D(x))%20#0) is:

#
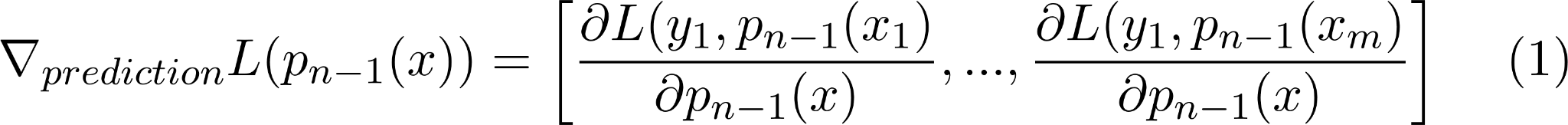


However XGBoost implements level-wise tree growth. The data is split into two nodes, where each node splits the data prioritizing the nodes closer to the tree root [2]. XGBoost splits up to the specified maximum depth parameter and the tree is pruned for branches in which there is little to no positive gain [2].

**Random Forest:**

Random Forest Classification is also a tree-based method. A number of individual decision trees operate as an ensemble. A dataset is split up into subsets and put into a tree. The training phases produce a prediction result based on each decision tree resulting in a prediction result [
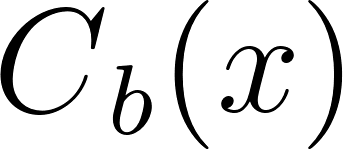
](https://www.codecogs.com/eqnedit.php?latex=%20C%7B_b%7D(x)%20#0). During the testing phase, the Random Forest classifier predicts the final decision of a new data point, noted by [
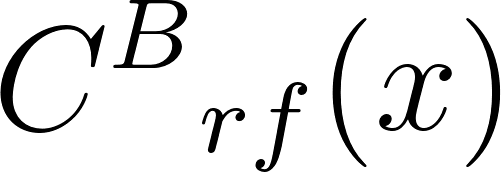
](https://www.codecogs.com/eqnedit.php?latex=%20%7BC%7D%7B%5EB%7D%7B_%7Brf%7D%7D(x)%20#0) using the majority vote of all [
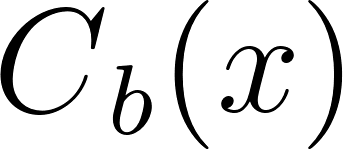
](https://www.codecogs.com/eqnedit.php?latex=%20%7BC%7D%7B_b%7D(x)%20#0) [3]. This yields the following:


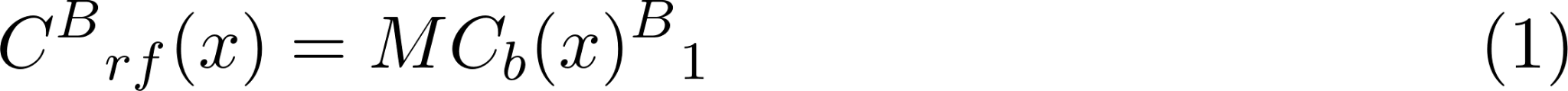


where [
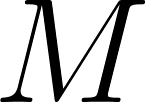
](https://www.codecogs.com/eqnedit.php?latex=%20M%20#0) =is the majority vote for an outcome. Out of all the decision-tree methods, Random Forest Classification is the method that is the most computationally expensive as it takes all possible combinations of subsets and puts those in a tree [3]. Without PCA, the computation cost will be significantly high.

**Ensemble Model Logistic Regression:**

The logistic regression model can be used on the dependent variables that are categorical in nature. It used to model the probability in which an outcome will occur. In our case the binary outcome is in the category <30 days. For any outcome 0 and 1 and assuming normalization parameters:

[
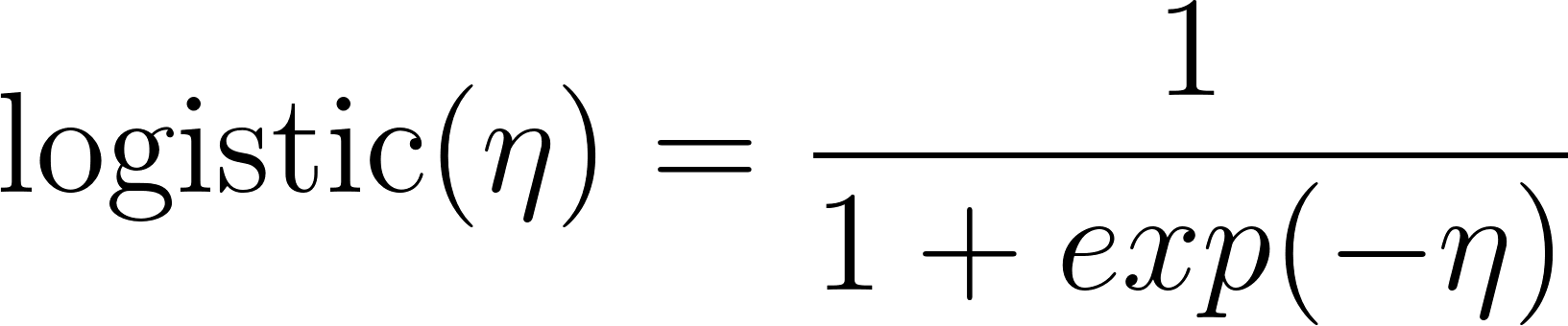
](https://www.codecogs.com/eqnedit.php?latex=%20%5Ctext%7Blogistic%7D(%5Ceta)%3D%5Cfrac%7B1%7D%7B1%2Bexp(-%5Ceta)%7D#0) [4]

Contrary to linear regression (often used for regression problems), instead of fitting a straight line or hyperplane, the logistic regression model uses the logistic function to produce the output of a linear equation that’s between 0 and 1. This model is very popular due to its simplicity and most importantly its interpretability - giving the user the ability to extract weights of each feature, or in other words, allow users to see what features the models deem as most important [5].

**Calculation for the RIW COSTs:**

The Resource Intensity Weight Value are the ratios that measure expected use of resources associated with different diagnostic, surgical procedure and demographic characteristics of an individual. The algorithm above shows how to obtain the RIW [6].
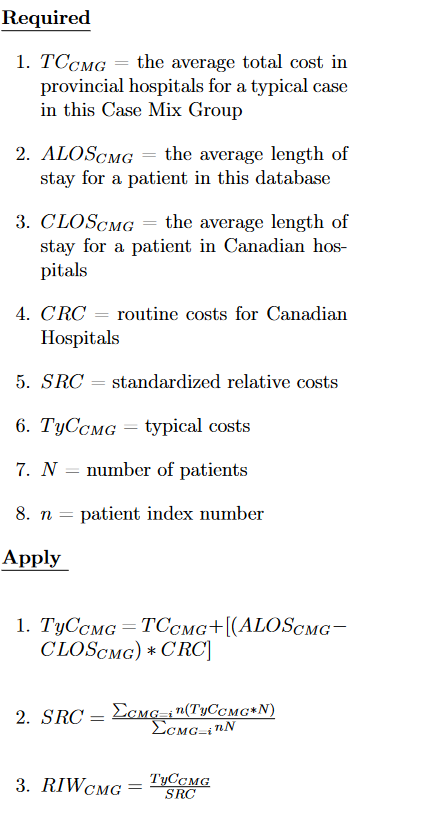


# Appendix B: Results

## Cumulative Variance For PCA (n = 65 components)


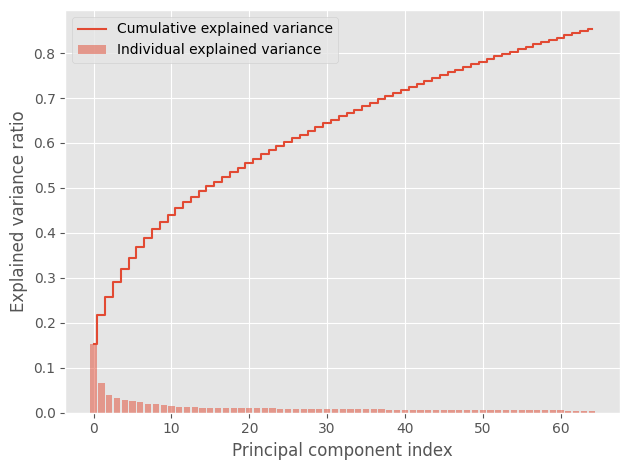


Figure [S1]: Cumulative Variances for All PCA Components (red line) and Individualized Variances (bar) for Most PCA components.

#

## Confusion Matrices

Confusion matrices were generated for both the general ensemble model and the individual submodels. These matrices were used to determine the performance of the classification problems by identifying the number of true positives (TP), false negatives (FN), false positives (FP), and true negatives (TN). TP represented the cases where the model correctly predicted a positive outcome, while FN represented the cases where the model incorrectly predicted a negative outcome. FP represented the cases where the model incorrectly predicted a positive outcome, while TN represented the cases where the model correctly predicted a negative outcome.


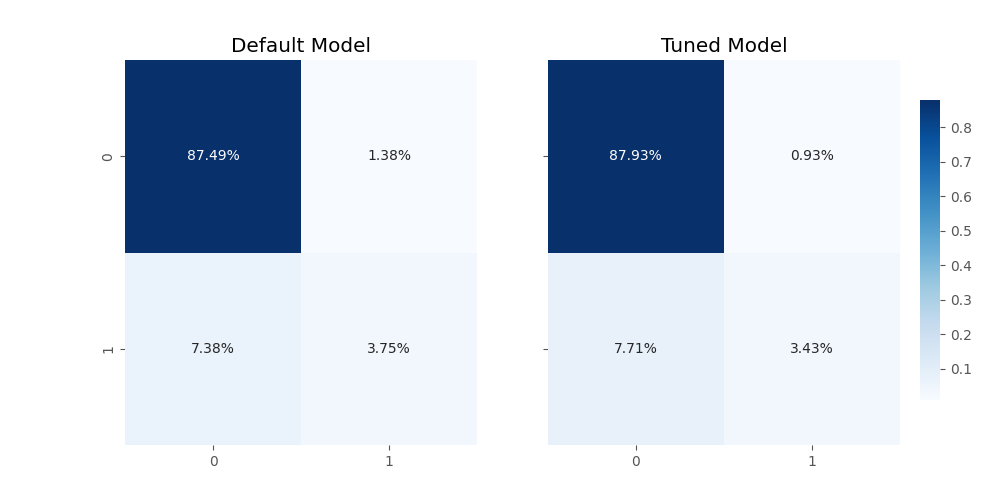


Figure [S2]: Confusion Matrices for the XGBoost Model with PCA.


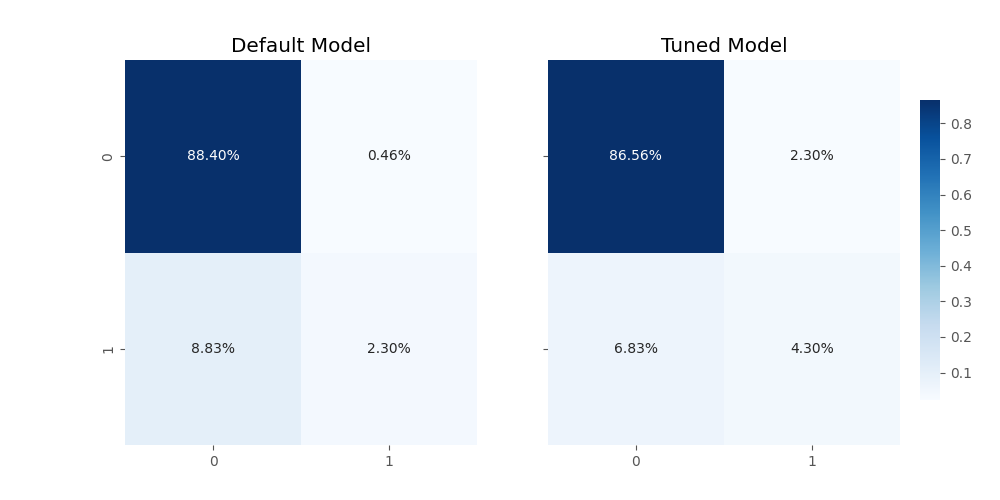


Figure [S3]: Confusion Matrices for the Random Forest Model with PCA


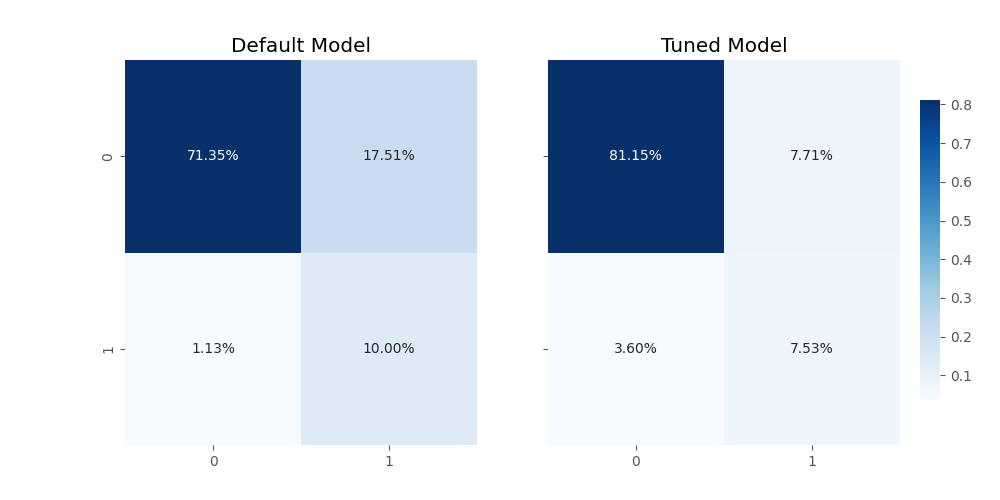


Figure [S4]: Confusion Matrices for LightGBM Model with PCA


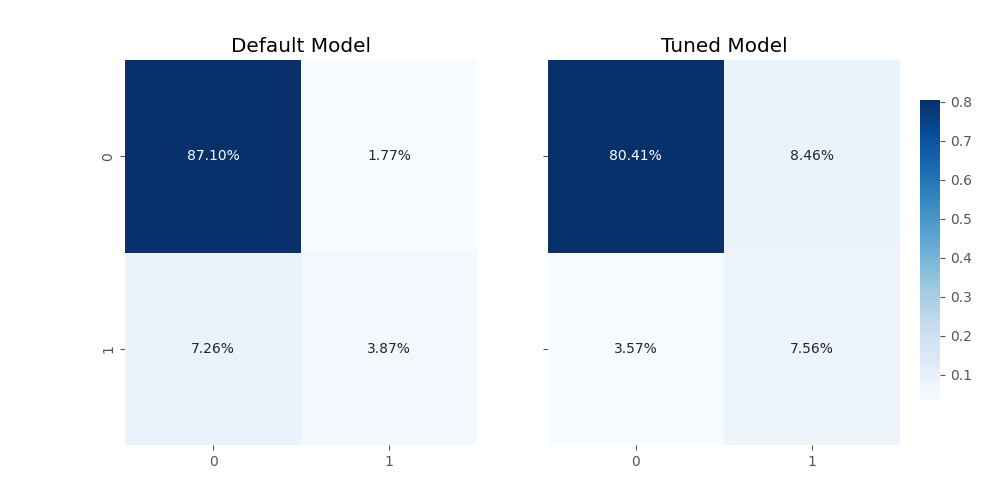


Figure [S5a]: Confusion Matrices for Ensemble Model with PCA. The matrices consists of the default base estimators with default (left) and tuned (right) logistic regression final estimator.


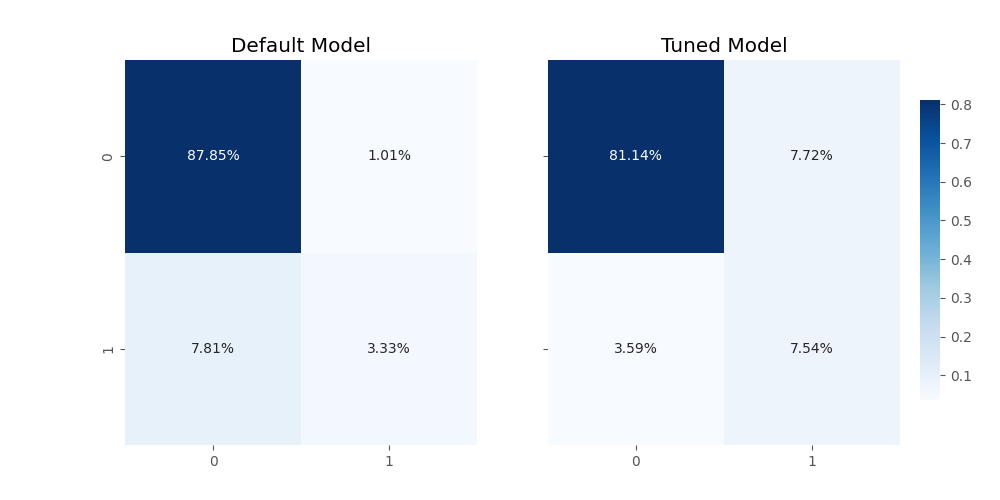


Figure [S5b]: Confusion Matrices for Ensemble Model with PCA. The matrices are ordered with the tuned base estimators with default (left) and tuned (right) logistic regression final estimator.

## Classification reports

Evaluation metrics for the ensemble model were presented using Classification Reports. In this context, class 0 represented the model's performance for the negative class (i.e., patients who did not return within 30 days), and class 1 represented the model's performance for the positive class (i.e., patients who did return within 30 days).

Table [S1]: Classification reports for different submodels^a^

| Model type | Class | Precision | Recall | *F*_1_-score |
| --- | --- | --- | --- | --- |
| **XGBoost** | | | | |
|  | 0^b^ | 0.92 | 0.98 | 0.95 |
|  | 1^c^ | 0.73 | 0.34 | 0.91 |
| **Random Forest** | | | | |
|  | 0 | 0.91 | 0.99 | 0.95 |
|  | 1 | 0.83 | 0.21 | 0.33 |
| **LightGBM** | | | | |
|  | 0 | 0.98 | 0.80 | 0.88 |
|  | 1 | 0.36 | 0.90 | 0.52 |

^a^All of these models have not been hyperparameter tuned.

^b^For all models, class 0 contains n=16,592.

^c^For all models, class 1 contains n=2079.

Table [S2]: Classification Reports for all The Ensemble Model Types

| Model type | Class | Precision | Recall | *F*_1_-score |
| --- | --- | --- | --- | --- |
| **Ensemble Model 1^a^** | | | | |
|  | 0^b^ | 0.92 | 0.98 | 0.95 |
|  | 1^c^ | 0.69 | 0.35 | 0.46 |
| **Ensemble Model 2^d^** | | | | |
|  | 0 | 0.96 | 0.90 | 0.93 |
|  | 1 | 0.47 | 0.68 | 0.56 |
| **Ensemble Model 3^e^** | | | | |
|  | 0 | 0.92 | 0.99 | 0.95 |
|  | 1 | 0.77 | 0.30 | 0.43 |

^a^Default Submodels and Default Logistic Regression

^b^For all models, class 0 contains n=16,592.

^c^For all models, class 1 contains n=2079.

^d^Default Submodels and Tuned Logistic Regression

^e^Tuned Submodels and Default Logistic Regression

## Linear Regression Plots

A least squares linear regression model was fitted on the expected length of stay and resource intensity weight value columns of a geographical dataset, and a summary of the best-fitted lines was obtained. The corresponding plot produced by the least squares linear regression was also obtained, and the results were separated by readmission status, age group, and gender. Note: The last row of each of the figures were shifted to the center and axes were included for 65-69 years using GIMP.


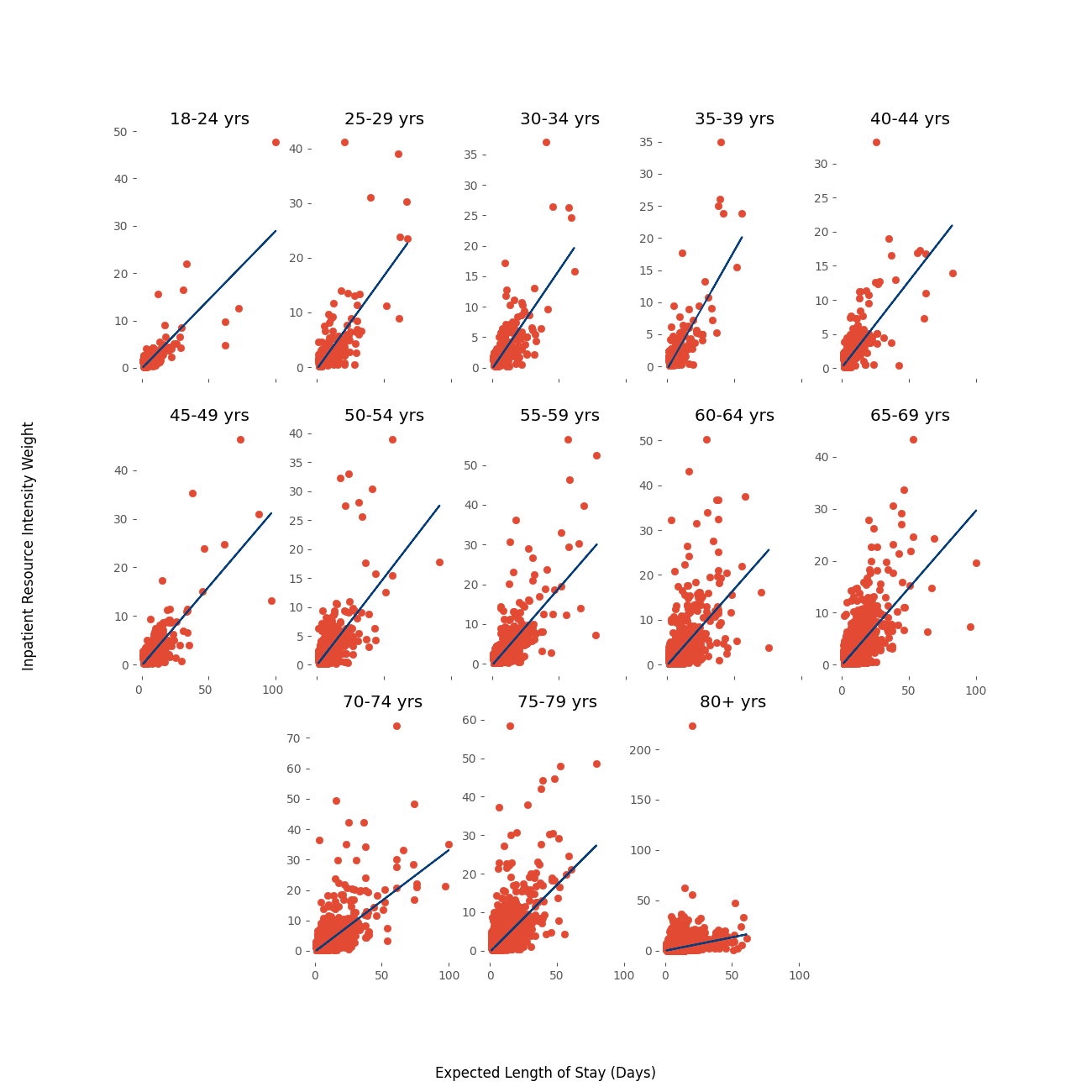


Figure [S6a]: Plot of regression line for women in all age groups who are not readmitted within 30 days.


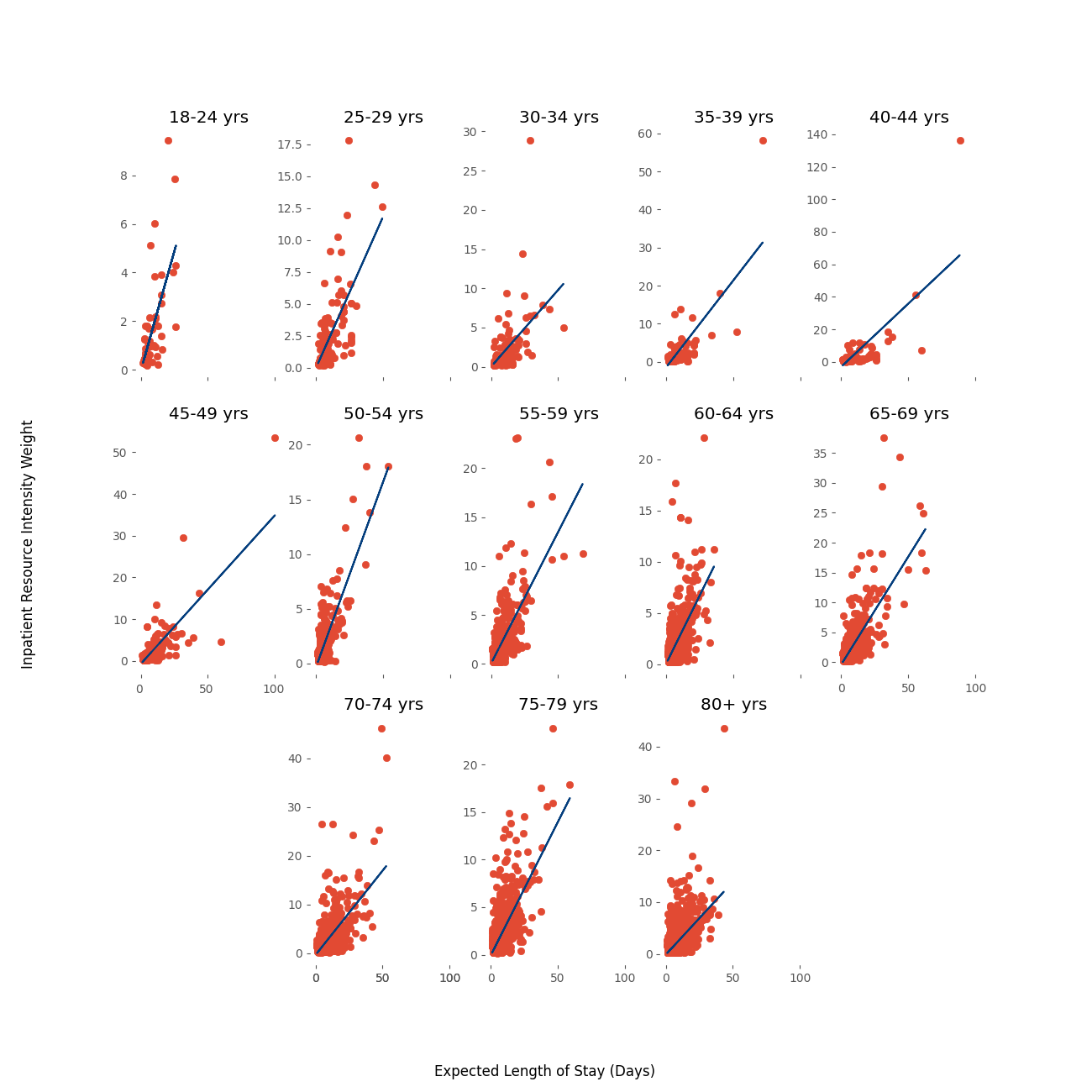


Figure [S6b]: Plot of regression line for women in all age groups who are readmitted within 30 days.


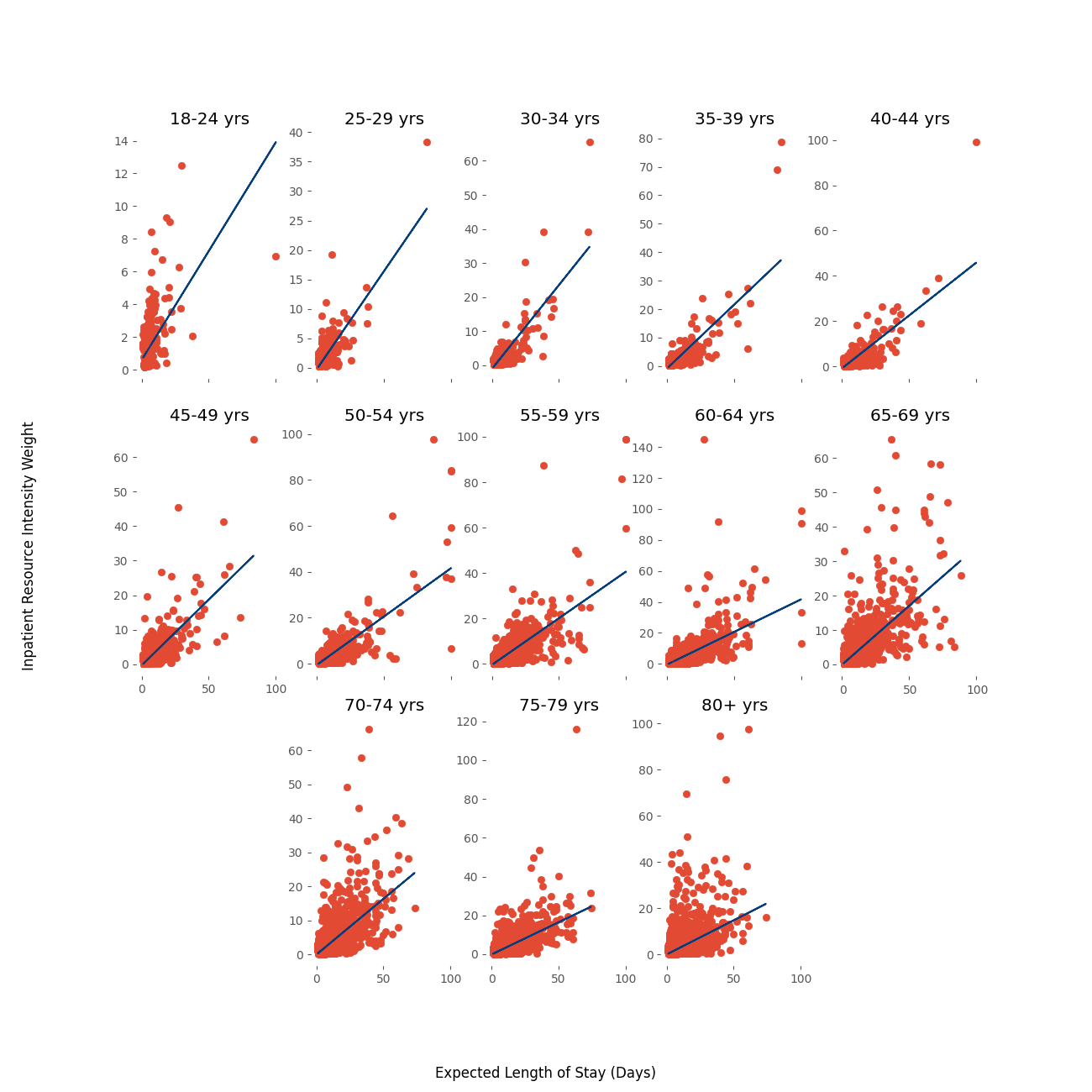


Figure [S7a]: Plot of regression line for men in all age groups who are not readmitted within 30 days.


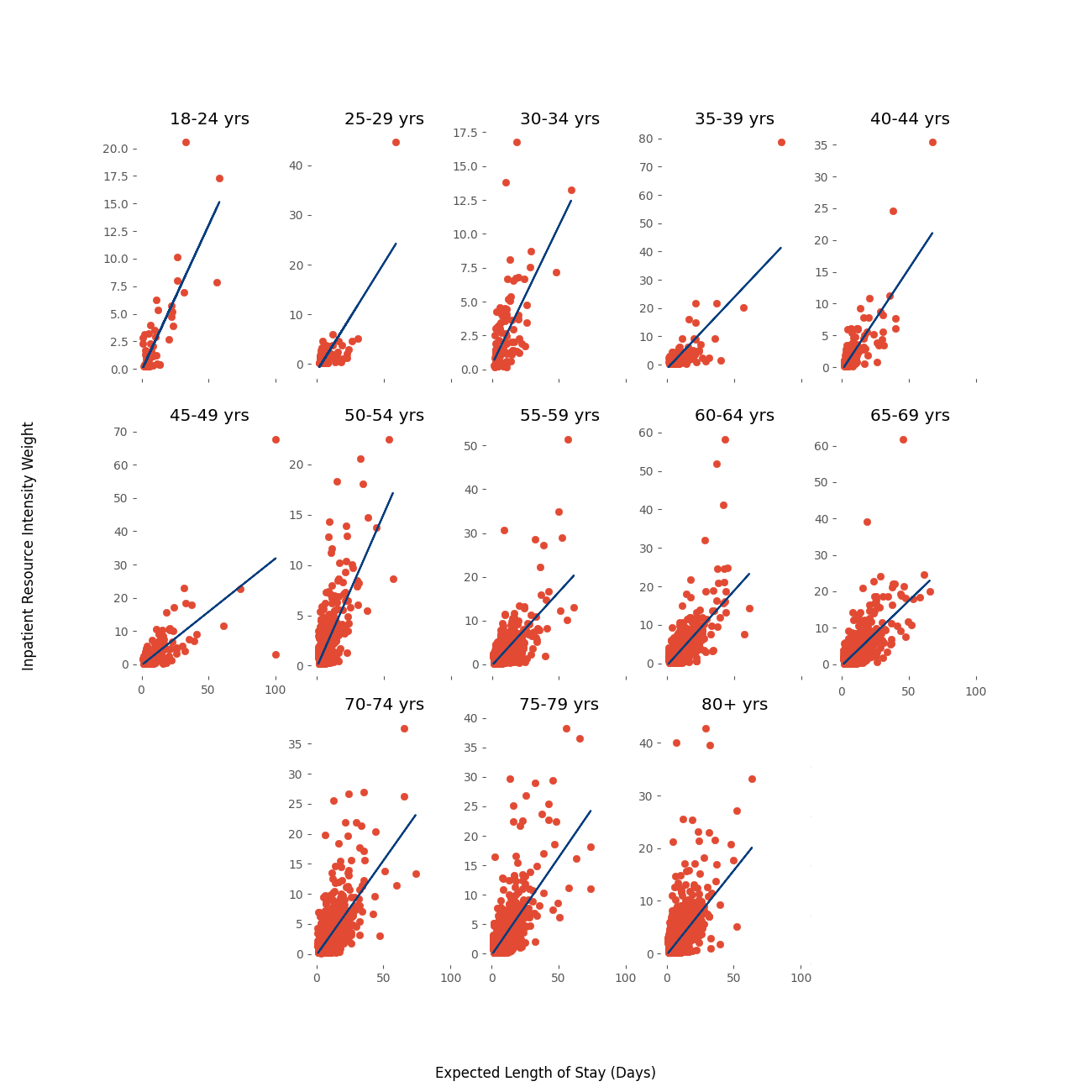


Figure [S7b]: Plot of regression line for men in all age groups who are readmitted within 30 days.

# Appendix C: Code

The main developers behind the python code were ER, KN, QG.

## Clinical Dataset Notebook

*# import necessary packages*
**from** datetime **import** datetime, timedelta
**import** numpy **as** np
**import** pandas **as** pd
**import** re
**import** time

***Formation One Dataset Via Isolation for Cardiovascular Diseases and Other Manipulations***

Here we will create a combined discharge abstract dataset allowing us to account for overall trends and not just the COVID-19 Datasets.

***Step 1.1: Obtain Data Sets***

As mentioned in the manuscript, we will be using the Discharge Abstract Data Set from:

- 2015-2016
- 2016-2017
- 2017-2019 (within a 24 month period 2017-2018 and 2018-2019)
- 2019-2021 (within a 24 month period 2019-2021 and 2020-2021)

First we will create several data frames for each of the individual datasets.

**import** pyreadstat

*# https://towardsdatascience.com/visualizing-clusters-with-pythons-matplolib-35ae03d87489*
*# first naming each of the data sets*
clin_1516, _ = pyreadstat.read_sav('../../data/clinical/1516.sav')
clin_1617, _ = pyreadstat.read_sav('../../data/clinical/1617.sav')
clin_1719, _ = pyreadstat.read_sav('../../data/clinical/1719.sav')
clin_1921, _ = pyreadstat.read_sav('../../data/clinical/1921.sav')

*# determining the dimensions of the datasets (number of rows and columns)*
display(clin_1516.shape, clin_1617.shape, clin_1719.shape, clin_1921.shape)

***Step 1.2: Analysis of Date Column***

As no reference date is provided, we assumed a reference date at random which was Jan 5th of a year (for example, 1516 would have a reference date of 2015-Jan-5th).

**def** get_date(row, date, type):
 *'''*
 *Arguments:*

 *row: Pandas Series - a single row of a DataFrame*
 *date: string - a date string in the format YYYY-MM-DD*
 *type: string - a string specifying whether the date represents an admission or a discharge date*

 *Purpose:*
 *calculate the absolute date based on a relative date and a reference date (either admission date or discharge date)*

 *Outputs:*
 *a Python datetime.date object that represents the absolute date calculated using the input relative date, reference date, and reference date type (admission or discharge).*


 *'''*
 **if** type.lower() == "discharge":
 relative_date = row['REL_DDAY']
 **elif** type.lower() == "admission":
 relative_date = row['REL_ADAY'] *# if admission is lower than discharge date then we can use the admission date as a*
 date_time_obj = datetime.strptime(date, "%Y-%m-%d").date() + timedelta(days = relative_date)

 **return** date_time_obj


**def** get_date_string(year):
 *"""*
 *Arguments:*

 *year: integer - a year in integer format*

 *Purpose:*
 *The purpose of this function is to construct a string representing a date of January 5th in the specified year.*

 *Output Description:*
 *This function returns a string in the format "YYYY-01-05" that represents the date of January 5th in the input year*

 *"""*
 **return** str(year) + "-" + "01-05"

clin_1921['ADMISSION_DATE'] = clin_1921.apply(**lambda** row: get_date(row, get_date_string(2019), "admission"), axis = 1)
clin_1921['DISCHARGE_DATE'] = clin_1921.apply(**lambda** row: get_date(row, get_date_string(2019), "discharge"), axis = 1)
clin_1719['ADMISSION_DATE'] = clin_1719.apply(**lambda** row: get_date(row, get_date_string(2017), "admission"), axis = 1)
clin_1719['DISCHARGE_DATE'] = clin_1719.apply(**lambda** row: get_date(row, get_date_string(2017), "discharge"), axis = 1)
clin_1617['ADMISSION_DATE'] = clin_1617.apply(**lambda** row: get_date(row, get_date_string(2016), "admission"), axis = 1)
clin_1617['DISCHARGE_DATE'] = clin_1617.apply(**lambda** row: get_date(row, get_date_string(2016), "discharge"), axis = 1)
clin_1516['ADMISSION_DATE'] = clin_1516.apply(**lambda** row: get_date(row, get_date_string(2015), "admission"), axis = 1)
clin_1516['DISCHARGE_DATE'] = clin_1516.apply(**lambda** row: get_date(row, get_date_string(2016), "discharge"), axis = 1)

***Step 1.3: Merging the Datasets***

Now that we have the admission dates we will create a merged dataset that takes into account all years.

*# combining the datasets*
clin_combined = pd.concat([clin_1516, clin_1617, clin_1719, clin_1921], ignore_index=True)

*# checking to see whether or not the number of columns have changed*
clin_combined.shape

Since the number of columns have not changed we can move on to the next part of our data reduction step.

***Step 1.4: Looking at Patients with Heart Diseases:***

The following ICD-10 codes are similar to Baurah, Liu et. al's models:

- I092 - Chronic rheumatic percarditis
- I098 - Other Specific rheumatic heart diseases
- I099 - Rheumatic heart Diseases
- I100 - Benign Hypertension
- I101 - Malignant Hypertension
- I11 - Hypertensive Heart Disease
- I13 - Hypertensive Heart and Renal Disease
- I500 - Congestive Heart Failure
- I501 - Left Ventricular Failure
- I509 - Heart Failure, Unspecified
- I516 - Cardiovascular Disease
- I518 - Other ill-defined heart disease
- I519 - Heart Disease, undefined
- I520 - Other heart disorders in bacterial diseases, classified elsewhere
- I521 - Other heart disorders in infectious diseases, classified elsewhere
- I528 - Other heart disorders in other diseases, classified elsewhere

We will be using these ICD-10-CODES. We will also be removing any age groups that are below 18 as we are only examining adults.

clin_combined = clin_combined[~clin_combined["AGRP_F_D"].isin(["newborn", "0 days to 11 months", "1-7 yrs", "8-12 yrs", "13-17 yrs"])]

heart = pd.DataFrame()
**for** i **in** range(1, 25):
 heart = heart.append(clin_combined[clin_combined["".join(["D_I10_", str(i)])].isin(["I092", "I098", "I099", "I100", "I101", "I11", "I13", "I500", "I501", "I509", "I519", "I520", "I521", "I528"])], ignore_index = True)

heart = heart.drop_duplicates(ignore_index = True).reset_index(drop = True)

***Step 1.5: Adding a readmission category***

Now we need to create a binary readmission category that determines yes or no for whether or not they will be readmitted within 30 days

**def** make_readmission_col(df):

 *"""*
 *Arguments:*

 *df: Pandas DataFrame - the DataFrame to be processed*

 *Purpose:*
 *Add two new columns to the input DataFrame that provide information about patient readmissions:*
 *'READMITTED' column indicates whether a patient has been readmitted during the time period represented by the DataFrame*
 *'DAYS_TO_NEXT_ADMISSION' column provides the number of days until the patient's next admission (if applicable)*

 *Output Description:*

 *Modifies the input DataFrame in place.*
 *Resulting DataFrame has same rows and columns as input, but with additional columns: 'READMITTED', 'DAYS_TO_NEXT_ADMISSION', and 'LTORET30Days' which indicates whether the patient is likely to be readmitted in the next 30 days.*
 *A message is printed indicating that the 'READMITTED' column has been added to the DataFrame.*
 *"""*
 df.sort_values(by = 'ADMISSION_DATE', inplace = True) *# sort by Admission date*
 df['READMITTED'] = df.groupby(['PATNT_ID'])['PATNT_ID'].transform('count')
 df['READMITTED'] = df['READMITTED'].map(**lambda** x: 1 **if** x > 1 **else** 0) *# column tells you if they've been readmitted period in the time period*
 *# this next column tells you the number of days until their next admission*
 df['DAYS_TO_NEXT_ADMISSION'] = df[df['READMITTED'] == 1].groupby(['PATNT_ID'])[['ADMISSION_DATE', 'DISCHARGE_DATE']].apply(**lambda** x: (x.shift(-1)['ADMISSION_DATE'] - x['DISCHARGE_DATE']).to_frame('new'))
 df['LTORET30Days'] = (df['DAYS_TO_NEXT_ADMISSION'] <= pd.Timedelta('30 days')).astype(int) *# tells you if they're going to be re-admitted in the next 30 days*
 print("You made a readmission column!")


make_readmission_col(heart)

***Clinical Preprocessing and PCA implementation***

The purpose of this section is to extrapolate the best attributes for our model allowing for accurate and proper feature selection for our ensemble models. First we will create a mapping function that encodes our variables from categorical to numerical and then we will be using sklearn's PCA models to ensure automation of the PCA.

Principle Component Analysis (PCA) is a statistical technique widely used by data scientists and researchers to identify hidden patterns and relationships in high-dimensional datasets. By transforming the original variables into a new set of uncorrelated variables, called principal components, PCA helps to simplify and visualize complex data. The principal components are ordered according to their explanatory power, with the first component accounting for the greatest amount of variation in the data, followed by the second and so on. PCA has numerous applications in fields such as finance, biology, and image processing, making it an important tool for understanding and analyzing large datasets.

Mathematically the orthogonal transformations involved in PCA use set size z-dimensional vectors of coefficients [
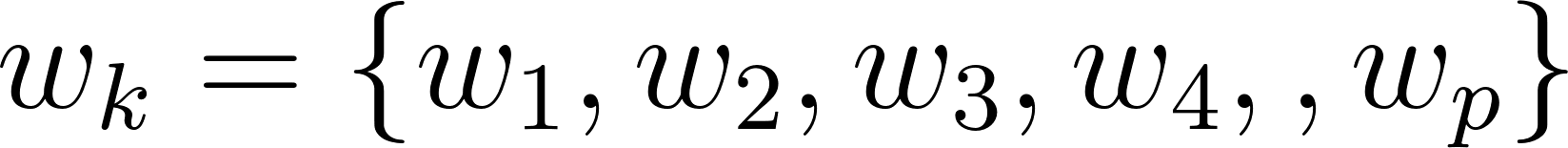
](https://latex-staging.easygenerator.com/eqneditor/editor.php?latex=w_k%20%3D%20%5C%7Bw_1%2C%20w_2%2C%20w_3%2C%20w_4%2C%20%E2%80%A6%2C%20w_p%5C%7D#0) associated with the mapping of each row vector [
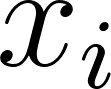
](https://www.codecogs.com/eqnedit.php?latex=x_i#0) to [
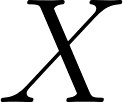
](https://www.codecogs.com/eqnedit.php?latex=X#0) to a new vector of principal component scores.

***Step 2.1: Creating an identical clone of the heart df and clinical preprocessing***

We will create a new copy of heart such that there is no need to start over again if we do eventually "mess up". We then transform the columns into number for easier handling, and deal with empty and null values.

diag1 = heart.copy()


*# Helper methods for mapping*
**def** map_mul_cul(df, cols):
 *"""*
 *Arguments:*

 *df: Pandas DataFrame - the DataFrame to be processed*
 *cols: list of strings - a list of column names to be processed*


 *Purpose:*

 *Convert categorical values in specified columns into numeric values*


 *Output Description:*

 *Modifies the input DataFrame in place*
 *Replaces categorical values in specified columns with numeric values*
 *Uses a dictionary to map between categorical values and their corresponding numeric values*


 *"""*
 v = list()
 **for** c **in** cols:
 df[c] = df[c].astype(str)
 v = np.unique(np.append(v, df[c]))
 vf = pd.factorize(v)
 vm = {vf[1][i]: vf[0][i] **for** i **in** range(len(vf[0]))}
 **for** c **in** cols:
 mapfxn(df, c, vm)


**def** build_col_names(base, num):
 *"""*
 *Generate a list of column names by concatenating a base string with integers.*

 *Args:*
 *base: A string that serves as the base of the column names.*
 *num: An integer that represents the number of column names to be generated.*

 *Returns:*
 *A list of strings where each string is a concatenation of the `base` argument*
 *and an integer ranging from 1 to `num`.*
 *"""*
 **return** [''.join([base, str(i)]) **for** i **in** range(1, num + 1)]


**def** map_lst(df, base, num):
 *"""*
 *Generate a list of column names and pass it to a function to perform some operation on a DataFrame.*

 *Args:*
 *df: A DataFrame object that will be operated on.*
 *base: A string that serves as the base of the column names.*
 *num: An integer that represents the number of column names to be generated.*

 *Returns:*
 *The output of the function `map_mul_cul` with two arguments:*
 *- `df`: the DataFrame object passed as an argument to `map_lst`*
 *- `build_col_names(base, num)`: a list of column names generated by `build_col_names` with the `base` and `num` arguments provided.*
 *"""*
 map_mul_cul(df, build_col_names(base, num))

*# Editing columns*
diag1 = diag1.drop(columns="GES_AGRP", axis='columns')
diag1 = diag1.drop(columns="WGHT_GRP", axis='columns')
diag1['ADMISSION_DATE'] = [dt.strftime('%Y%m%d') **for** dt **in** diag1['ADMISSION_DATE']]
diag1['DISCHARGE_DATE'] = [dt.strftime('%Y%m%d') **for** dt **in** diag1['DISCHARGE_DATE']]
diag1['DAYS_TO_NEXT_ADMISSION'] = [''.join(re.findall(r'\d+', str(d))) **if** str(d) != 'NaN' **else** -1 **for** d **in** diag1['DAYS_TO_NEXT_ADMISSION']]
*# These are the mappers that create a categorical label for each category that we are interested in:*

*# These are the mappers that create a categorical label for each category that we are interested in:*
province_mapper = {'': 0, '0': 0, '1': 1, '2': 2, '3': 3, '4': 4, '5': 5, '6': 6, '7': 7, '8': 8, 'T': 10}
age_levels_mapper = {'newborn': 1, '0 days to 11 months': 2, '1-7 yrs': 3, '8-12 yrs': 4, '13-17 yrs': 5, '18-24 yrs': 6, '25-29 yrs': 7, '30-34 yrs': 8, '35-39 yrs': 9, '40-44 yrs': 10, '45-49 yrs': 11, '50-54 yrs': 12,'55-59 yrs': 13, '60-64 yrs': 14, '65-69 yrs': 15, '70-74 yrs': 16, '75-79 yrs': 17, '80+ yrs': 20}
gender_mapper = {'M': 1, 'F': 2}
from_transfer_mapper = to_transfer_mapper = {'': 1, '0': 1, '6': 2, '1': 2, '9': 2, '4': 2, 'N': 2 , '8': 2, 'E': 2, '2': 2, 'A': 2, '3': 2, '5': 2, '2': 2 , '7': 2, 'G': 2, 'U': 2, 'M': 2, 'T': 2, 'C': 2, 'H': 2, 'F': 2, 'P': 2, 'S': 2, 'J': 2} *# 1 = no, # 2 = yes*
admit_mapper= {'': 0, 'L': 0, 'N':1, 'U': 2}
entry_mapper = {'': 0, 'E': 1, 'D': 2, 'N': 3, 'C': 4, 'P': 5}
discharge_deposition_score= {'': 0, '04': 4, '05': 4, '10': 3, '20': 3, '30': 3, '40': 3, '90': 3, '61': 2, '62': 2, '65': 2, '73': 1, '08': 0, '09': 0, '66': 0, '67': 0, '72': 0, '74': 0} *# this is utility gained that organizes this from utility gained from the discharge deposition*
ante_mapper = {'': 0, '0': 0, '1': 1, '2': 2, '3': 3, '4': 4, '5': 5, '6': 6, '7': 7, '8': 8, '9': 9, 'C': 11, 'U': 12}
special_care_unit_number = {'': 18,'10': 1, '20': 2, '25': 3, '30': 4, '35': 5, '40': 6, '45': 7, '50': 8, '51': 9, '52': 10, '53': 11, '60': 12, '70': 13, '80': 14, '90': 15, '95': 16, '98': 17, '99': 18}
diag_type_mapper = {'': 99, '0': 0, '1': 1, '2': 2, '3': 3, '4': 4, '5': 5, '6': 6, '7': 7, '8': 8, '9': 9, 'M': 10, 'W': 11, 'X': 12, 'Y': 13, 'Z': 14}


**def** mapfxn(df1, col, mp):
 *"""*
 *Replace values in a specified column of a DataFrame with their corresponding replacement values.*

 *Args:*
 *df1: A pandas DataFrame object to be operated on.*
 *col: A string representing the name of the column in `df1` on which the `replace` operation will be performed.*
 *mp: A dictionary or a pandas Series, where the keys represent the values in `col` to be replaced*
 *and the values represent the replacement values.*

 *Returns:*
 *None. The function modifies the DataFrame `df1` directly by replacing values in the specified column*
 *with their corresponding replacement values from the dictionary `mp`.*
 *"""*
 df1[col] = df1[col].replace(mp)


mapfxn(diag1, "SUB_PROV", province_mapper)
mapfxn(diag1, "AGRP_F_D", age_levels_mapper)
mapfxn(diag1, "X_FR_I_T", from_transfer_mapper)
mapfxn(diag1, "GENDER", gender_mapper)
mapfxn(diag1, "ADM_CAT", admit_mapper)
mapfxn(diag1, "ENT_CODE", entry_mapper)
mapfxn(diag1, "X_TO_I_T", to_transfer_mapper)
mapfxn(diag1, "DIS_DISP", discharge_deposition_score)

**for** i **in** range(1, 25):
 mapfxn(diag1, "".join(["D_TYP_", str(i)]), diag_type_mapper)

map_lst(diag1, 'D_I10_', 25)
map_lst(diag1, 'I_CCI_', 20)
map_lst(diag1, 'ST_AT_', 20)
map_lst(diag1, 'LC_AT_', 20)
map_lst(diag1, 'AN_TE_', 20)
map_lst(diag1, 'SCU_C_', 6)
map_lst(diag1, 'S_L_HR_', 6)

*# Managing empty/invalid values*
diag1 = diag1.replace(r'^\s*$', -1, regex=True)
diag1 = diag1.replace(np.NaN, -1)

***Step 2.2 PCA***

We conduct PCA to reduce the number of columns in our training data.

npca = diag1.copy()
spaca = diag1.copy()

**import** matplotlib.pyplot **as** plt
**from** sklearn.decomposition **import** PCA
**from** sklearn.model_selection **import** train_test_split
**from** sklearn.preprocessing **import** StandardScaler


*# implement regular linear PCA*
*# Split the data into test and train sets*
X = npca.drop(columns = ["LTORET30Days"])
y = npca["LTORET30Days"]
X_train, X_test, y_train, y_test = train_test_split(X, y, test_size=0.2, random_state=42)

*# Perform standard scalar normalization to normalize our feature set*
sc = StandardScaler()
X_train = sc.fit_transform(X_train)
X_test = sc.transform(X_test)

pca = PCA(n_components=65)
X_train = pca.fit_transform(X_train)
X_test = pca.transform(X_test)
exp_var_pca = pca.explained_variance_ratio_
cum_sum_eigenvalues = np.cumsum(exp_var_pca)

plt.style.use('ggplot')
plt.bar(range(0,len(exp_var_pca)), exp_var_pca, alpha=0.5, align='center', label='Individual explained variance')
plt.step(range(0,len(cum_sum_eigenvalues)), cum_sum_eigenvalues, where='mid',label='Cumulative explained variance')
plt.ylabel('Explained variance ratio')
plt.xlabel('Principal component index')
plt.legend(loc='best')
plt.tight_layout()
plt.show()

**def** myplot(score,coeff,y,labels=None):
 xs = score[:,0]
 ys = score[:,1]
 n = coeff.shape[0]
 scalex = 1.0/(xs.max() - xs.min())
 scaley = 1.0/(ys.max() - ys.min())
 plt.scatter(xs * scalex,ys * scaley, c=y)
 **for** i **in** range(n):
 plt.arrow(0, 0, coeff[i,0], coeff[i,1],color = 'r',alpha = 0.5)
 **if** labels **is** None:
 plt.text(coeff[i,0]* 1.15, coeff[i,1] * 1.15, "Var"+str(i+1), color = 'g', ha = 'center', va = 'center')
 **else**:
 plt.text(coeff[i,0]* 1.15, coeff[i,1] * 1.15, labels[i], color = 'g', ha = 'center', va = 'center')
 plt.xlim(-1,1)
 plt.ylim(-1,1)
 plt.xlabel("PC{}".format(1))
 plt.ylabel("PC{}".format(2))
 plt.grid()


indices = [i **for** i, name **in** enumerate(pca.get_feature_names_out()) **if** name.startswith('D_I10_')]
myplot(X_train[:, 0:2], np.transpose(pca.components_[0:2, indices]),y_train)
plt.show()

print(f'# Readmitted: {sum(y)}')
print(f'# Not Readmitted: {len(y) - sum(y)}')

Model Training and Hyperparameter Tuning

Setup

**from** datetime **import** timedelta
**from** scipy.stats **import** randint **as** sp_randint
**from** scipy.stats **import** uniform **as** sp_uniform
**import** seaborn **as** sns
**from** sklearn.metrics **import** (
 classification_report,
 confusion_matrix,
 ConfusionMatrixDisplay,
 f1_score,
 precision_score,
 recall_score,
 roc_auc_score,
)
**from** sklearn.model_selection **import** GridSearchCV, RandomizedSearchCV, RepeatedStratifiedKFold


X_train = X_train.copy()
y_train = y_train.copy()


**def** train_model(model):
 start_time = time.time()
 print("---- Starting training ----")
 model.fit(X_train, y_train)
 print(f"------ {timedelta(seconds = time.time() - start_time)} ------")


**def** eval_model(model):
 y_pred = model.predict(X_test)

 print()
 print()
 print()
 print("Summary")
 print(classification_report(y_test, y_pred))
 print('Confusion Matrix: ', confusion_matrix(y_test, y_pred))
 print('ROCAUC score: ', roc_auc_score(y_test, y_pred))
 print('F1 Score: ', f1_score(y_test, y_pred))
 print('Recall: ', recall_score(y_test, y_pred))
 print('Precision: ', precision_score(y_test, y_pred))


**def** train_and_tune_model(base_model, tuning_search):
 print("Base Model")
 print("----------------------------------------------------------------------------------------")
 train_model(base_model)
 eval_model(base_model)
 print("----------------------------------------------------------------------------------------")

 print()
 print()
 print()
 print("Tuned Model")
 print("----------------------------------------------------------------------------------------")
 train_model(tuning_search)
 print(tuning_search.best_params_)
 print(tuning_search.best_score_)
 eval_model(tuning_search)
 print("----------------------------------------------------------------------------------------")


**def** cm_fig(base_model, tuned_model, model_name):
 base_pred = base_model.predict(X_test)
 base_report = classification_report(y_test, base_pred, output_dict=True)
 base_cm = confusion_matrix(y_test, base_pred, normalize='all')

 tuned_pred = tuned_model.predict(X_test)
 tuned_report = classification_report(y_test, tuned_pred, output_dict=True)
 tuned_cm = confusion_matrix(y_test, tuned_pred, normalize='all')

 fig, axes = plt.subplots(1, 2, figsize=(10, 5), sharex=True, sharey=True)
 sns.heatmap(base_cm, ax=axes[0], cmap=plt.cm.Blues, annot=True, fmt=".2%", cbar=False)
 axes[0].set_title('Default Model')

 sns.heatmap(tuned_cm, ax=axes[1], cmap=plt.cm.Blues, annot=True, fmt=".2%", cbar_ax=fig.add_axes([.92, .2, .02, .6]))
 axes[1].set_title('Tuned Model')

 fig.savefig(f"./{model_name}_cm.png")

 base_df = pd.DataFrame(base_report).transpose()
 base_df.to_csv(f"{model_name}_base_report.csv")
 tuned_df = pd.DataFrame(tuned_report).transpose()
 tuned_df.to_csv(f"{model_name}_tuned_report.csv")

 bar_cr(base_df, f'Default {model_name} Model', f'{model_name}_base_bar.png')
 bar_cr(tuned_df, f'Tuned {model_name} Model', f'{model_name}_tuned_bar.png')


**def** bar_cr(df, title, file_name):
 metrics = ('precision', 'recall', 'f1')
 values = {
 'Not Readmitted': list(df.loc['0', ['precision', 'recall', 'f1-score']]),
 'Readmitted': list(df.loc['1', ['precision', 'recall', 'f1-score']]),
 }

 x = np.arange(len(metrics))
 bar_width = 0.25
 multiplier = 0

 plt.style.use('ggplot')
 fig, ax = plt.subplots()

 **for** grp, val **in** values.items():
 offset = bar_width*multiplier
 bars = ax.bar(x + offset, val, bar_width, label=grp, color=['#91d9fa' **if** grp == 'Readmitted' **else** '#ff7c70'])
 multiplier += 1

 ax.set_ylabel('Score')
 ax.set_title(title)
 ax.set_xticks(x + bar_width/2, metrics)
 ax.legend(loc='upper right', ncol=1, frameon=True)
 ax.set_ylim(0, 1.2)

 plt.show()

 fig.savefig(file_name)

XGB

**from** xgboost **import** XGBClassifier

base_xgb = XGBClassifier()

params_xgb = {
 'max_depth': range(2, 10, 4),
 'n_estimators': range(60, 240, 60),
 'learning_rate': [0.1, 0.01, 0.05]
}

tuned_xgb = GridSearchCV(
 estimator = base_xgb,
 param_grid = params_xgb,
 scoring = "recall",
 cv = 3,
 n_jobs = -1,
 verbose = 4,
 error_score = 'raise'
)

train_and_tune_model(base_xgb, tuned_xgb)
cm_fig(base_xgb, tuned_xgb, 'XGB')

Random Forest Classifier

**from** sklearn.ensemble **import** RandomForestClassifier

base_rf = RandomForestClassifier(class_weight='balanced', verbose=True)

params_rf = {
 'bootstrap': [True, False],
 'max_depth': [40, 120, None],
 'max_features': ['sqrt'],
 'min_samples_leaf': [4],
 'min_samples_split': [10],
 'n_estimators': [1200]
}

tuned_rf = GridSearchCV(
 estimator = base_rf,
 param_grid = params_rf,
 scoring = 'f1',
 cv = 3,
 verbose = 4,
 n_jobs = -1,
 error_score = 'raise'
)

train_and_tune_model(base_rf, tuned_rf)
cm_fig(base_rf, tuned_rf, 'RF')

LGBM

**from** lightgbm.sklearn **import** LGBMClassifier

base_lgbm = LGBMClassifier(random_state=42, class_weight='balanced', verbose=4)

params_lgbm = {
 'learning_rate' : [0.01, 0.02, 0.03, 0.04, 0.05, 0.08, 0.1, 0.2, 0.3, 0.4],
 'n_estimators' : [100, 200, 300, 400, 500, 600, 800, 1000, 1500, 2000],
 'num_leaves': sp_randint(6, 50),
 'min_child_samples': sp_randint(100, 500),
 'subsample': sp_uniform(loc = 0.2, scale = 0.8),
 'max_depth': [-1, 1, 2, 3, 4, 5, 6, 7],
 'colsample_bytree': sp_uniform(loc = 0.4, scale = 0.6),
 'reg_alpha': [0, 1e-1, 1, 2, 5, 7, 10, 50, 100],
 'reg_lambda': [0, 1e-1, 1, 5, 10, 20, 50, 100],
 'min_data_in_leaf': sp_randint(20, 200),
}

tuned_lgbm = RandomizedSearchCV(
 estimator = base_lgbm,
 param_distributions = params_lgbm,
 n_jobs = -1,
 n_iter = 500,
 scoring = 'f1',
 cv = RepeatedStratifiedKFold(n_splits=2, n_repeats=2, random_state=42),
 refit = True,
 random_state = 42,
 verbose = 3,
 error_score = 'raise'
)

train_and_tune_model(base_lgbm, tuned_lgbm)
cm_fig(base_lgbm, tuned_lgbm, 'LGBM')

Ensemble Model

**from** lightgbm.sklearn **import** LGBMClassifier
**from** sklearn.ensemble **import** RandomForestClassifier
**from** sklearn.ensemble **import** StackingClassifier
**from** sklearn.linear_model **import** LogisticRegression
**from** sklearn.svm **import** SVC
**from** xgboost **import** XGBClassifier

base_classifiers = [
 ('xgb', base_xgb),
 ('rf', base_rf),
 ('lgbm', base_lgbm)
]

base_stacking_model = StackingClassifier(base_classifiers, cv='prefit', final_estimator = LogisticRegression(class_weight='balanced'))

tuned_classifiers = [
 ('xgb', tuned_xgb),
 ('rf', tuned_rf),
 ('lgbm', tuned_lgbm)
]

base_tuned_stacking_model = StackingClassifier(tuned_classifiers, cv='prefit', final_estimator = LogisticRegression(class_weight='balanced'))

train_model(base_stacking_model)
eval_model(base_stacking_model)

train_model(base_tuned_stacking_model)
eval_model(base_tuned_stacking_model)

**import** itertools

**def** gscv_stacking_lr(bc):
 pp = {
 'final_estimator__solver': ['sag'],
 'final_estimator__penalty': ['l2'],
 'final_estimator__C': np.logspace(0, -9, num=200)
 }

 best_params_ = []
 best_score_ = 0
 best_model = None

 param_pers = list(itertools.product(*pp.values()))

 print(f'Creating {len(param_pers)} fits')
 count = 0

 **for** params **in** param_pers:
 count += 1
 print(f'Fit #{count} of {len(param_pers)}')

 p = {'solver': params[0], 'penalty': params[1], 'C': params[2]}
 model = StackingClassifier(
 bc,
 cv='prefit',
 final_estimator = LogisticRegression(class_weight='balanced', **p)
 )

 **try**:
 train_model(model)
 **except**:
 print('Fail')
 **continue**

 y_pred = model.predict(X_test)
 f1 = f1_score(y_test, y_pred)
 **if** f1 > best_score_:
 best_score_ = f1
 best_params_ = p
 best_model = model

 print(best_params_)
 print(best_score_)

 **return** best_params_, best_score_, best_model

p, s, bctf = gscv_stacking_lr(base_classifiers)

gnb_stacking_model = StackingClassifier(
 tuned_classifiers,
 cv='prefit',
 final_estimator = LogisticRegression(class_weight='balanced', **{'solver': 'liblinear', 'penalty': 'l2', 'C': 0.00042919342601287783})
)

train_model(gnb_stacking_model)
eval_model(gnb_stacking_model)

cm_fig(base_stacking_model, bctf, 'LR Default SM Ensemble')
cm_fig(base_tuned_stacking_model, gnb_stacking_model, 'LR Tuned SM Ensemble')

cm_fig(base_tuned_stacking_model, gnb_stacking_model, 'Ensemble')

## Geo Dataset Notebook

Formation of Two Datasets Via Extraction and Isolation for two Datasets According to Diagnosis Type

Here we will be creating a combined geo discharge abstract database allowing us to account for overall trends and not just the COVID-19 dataset.

*# Import necessary Packages:*
**import** pyreadstat
**import** pandas **as** pd
**import** numpy **as** np
**from** datetime **import** datetime, timedelta
**import** io
**import** requests
**import** warnings
warnings.simplefilter('ignore')
**from** sklearn **import** preprocessing
**import** numpy **as** np
**from** sklearn.decomposition **import** PCA
**from** sklearn.cluster **import** KMeans
**from** sklearn.preprocessing **import** OneHotEncoder, StandardScaler
**from** scipy.sparse **import** hstack
**from** sklearn **import** svm
**from** sklearn.preprocessing **import** OneHotEncoder
**from** sklearn.model_selection **import** train_test_split
**from** sklearn.feature_selection **import** SelectKBest
**from** sklearn.feature_selection **import** chi2
**import** seaborn **as** sns
**import** matplotlib.pyplot **as** plt
**from** scipy.stats **import** ttest_ind
**from** sklearn.linear_model **import** LinearRegression
**from** scipy.stats **import** gaussian_kde

Step 1.1: Obtain Data Sets

As mentioned in the manuscript, we will be using the Discharge Abstract Data Set from:

- 2015-2016
- 2016-2017
- 2017-2019 (within a 24 month period 2017-2018 and 2018-2019)
- 2019-2021 (within a 24 month period 2019-2021 and 2020-2021)

First we will create several dataframes for each of the individual datasets.

*#first naming each of the data sets*
geo_1516, _ = pyreadstat.read_sav('../../data/geo/1516.sav')
geo_1617, _ = pyreadstat.read_sav('../../data/geo/1617.sav')
geo_1719, _ = pyreadstat.read_sav('../../data/geo/1719.sav')
geo_1921, _ = pyreadstat.read_sav('../../data/geo/1921.sav')

*#determining the dimensions of the datasets (number of rows and columns)*
display(geo_1516.shape, geo_1617.shape, geo_1719.shape, geo_1921.shape)

Step 1.4: Analysis of the Date Column

We will be using the same method as in the clinical dataset.

**def** get_date(row, date, type):
 *'''*
 *data will be a string in the format YYYY-MM-DD*
 *'''*
 **if** type.lower() == "discharge":
 relative_date = row['REL_DDAY']
 **elif** type.lower() == "admission":
 relative_date = row['REL_ADAY'] *# if admission is lower than discharge date then we can use the admission date as a*
 date_time_obj = datetime.strptime(date, "%Y-%m-%d").date() + timedelta(days = relative_date)

 **return** date_time_obj

**def** get_date_string(year):
 **return** str(year) + "-" + "01-05"

geo_1921['ADMISSION_DATE'] = geo_1921.apply(**lambda** row: get_date(row, get_date_string(2019), "admission"), axis = 1)
geo_1921['DISCHARGE_DATE'] = geo_1921.apply(**lambda** row: get_date(row, get_date_string(2019), "discharge"), axis = 1)
geo_1719['ADMISSION_DATE'] = geo_1719.apply(**lambda** row: get_date(row, get_date_string(2017), "admission"), axis = 1)
geo_1719['DISCHARGE_DATE'] = geo_1719.apply(**lambda** row: get_date(row, get_date_string(2017), "discharge"), axis = 1)
geo_1617['ADMISSION_DATE'] = geo_1617.apply(**lambda** row: get_date(row, get_date_string(2016), "admission"), axis = 1)
geo_1617['DISCHARGE_DATE'] = geo_1617.apply(**lambda** row: get_date(row, get_date_string(2016), "discharge"), axis = 1)
geo_1516['ADMISSION_DATE'] = geo_1516.apply(**lambda** row: get_date(row, get_date_string(2015), "admission"), axis = 1)
geo_1516['DISCHARGE_DATE'] = geo_1516.apply(**lambda** row: get_date(row, get_date_string(2015), "discharge"), axis = 1)

Step 1.3: Merging the Datasets

We will now combine the geo_dataset into one big dataset. Here we do not worry about the admission dates as we need the relative discharge day only and total days spent in the hospitals as our main quantity.

geo_combined = pd.concat([geo_1516, geo_1617, geo_1719, geo_1921], ignore_index=True)
geo_combined.shape
geo_combined.columns

Step 1.5: Adding a readmission category

Now we need to create a binary readmission category that determines yes or no for whether or not they were readmitted within 30 days.

**def** make_readmission_col(df):
 df.sort_values(by = 'ADMISSION_DATE', inplace = True) *# sort by Admission date*
 df['READMITTED'] = df.groupby(['PATNT_ID'])['PATNT_ID'].transform('count')
 df['READMITTED'] = df['READMITTED'].map(**lambda** x: 1 **if** x > 1 **else** 0) *# column tells you if they've been readmitted period in the time period*
 *# this next column tells you the number of days until their next admission*
 df['DAYS_TO_NEXT_ADMISSION'] = df[df['READMITTED'] == 1].groupby(['PATNT_ID'])[['ADMISSION_DATE', 'DISCHARGE_DATE']].apply(**lambda** x: (x.shift(-1)['ADMISSION_DATE'] - x['DISCHARGE_DATE']).to_frame('new'))
 df['LTORET30Days'] = (df['DAYS_TO_NEXT_ADMISSION'] <= pd.Timedelta('30 days')).astype(int) *# tells you if they're going to be re-admitted in the next 30 days*
 **return** print("You made a readmission column!")
make_readmission_col(geo_combined)

Step 1.5: Seperation of data set into groups:

We will now perform the following isolations and seperations:

- Isolation of the age groups: We will be considering only Canadian adults as newborn hospital readmissions are quite difficult to predict.
- Isolation of the CMG and MCC groups: in this case we want to ensure that our RIW value is not affected by any confounding variables: We will be isolating for only the groups whose values are 5. This allows us to focus on the patients who have a Card & Vasc Diseases Circ System.
- Isolation of Males and Females
- Splitting into those who have been readmitted and those who have not been readmitted.

gc = geo_combined[geo_combined['MCC_CODE']=='05']
gc_f =gc[~gc["AGRP_F_D"].isin(["newborn", "0 days to 11 months", "1-7 yrs", "8-12 yrs", "13-17 yrs"])]
gc_M = gc_f[gc_f["GENDER"]=='M']
gc_F = gc_f[gc_f["GENDER"]=='F']

gc_M1 = gc_M[gc_M['LTORET30Days']==0]
gc_M2 = gc_M[gc_M['LTORET30Days']==1]
gc_F1 = gc_F[gc_F['LTORET30Days']==0]
gc_F2 = gc_F[gc_F['LTORET30Days']==1]

*# creates a function that*
**def** sortdict(myDict):
 *"""*
 *Args:*
 *-Takes a dictionary*
 *Outputs:*
 *-Ordered Dictionary*
 *"""*
 myKeys = list(myDict.keys())
 myKeys.sort()
 sorted_dict = {i: myDict[i] **for** i **in** myKeys}
 **return** sorted_dict

**def** spdba(df):
 *"""*
 *Args:*
 *-Takes a dataframe and groups them to the column age_range*
 *Outputs:*
 *a couple of new grouped dataframes with name df_{age range} as a dictionary that is sorted.*
 *"""*
 ages = set(df.loc[:, 'AGRP_F_D'])
 grps = df.groupby(['AGRP_F_D'])
 grpd = {}
 **for** age **in** ages:
 grpd[age] = grps.get_group(age)
 grpd = sortdict(grpd)
 **return** grpd

M1 = spdba(gc_M1)
M2 = spdba(gc_M2)
F1 = spdba(gc_F1)
F2 = spdba(gc_F2)

Step 2: Performing a Mutliple Linear Regression Analysis

Our algorithm performs a linear regression to determine an approximate relationship between the ELOS and RIW.

**import** statsmodels.api **as** sm

**def** linear_regression(df, x_col, y_col):
 *"""*
 *Arguments: Takes dataframe, x_col, y_col*
 *Outputs: linear regression, equation with summary statistics.*
 *"""*
 x = df[x_col]
 y = df[y_col]
 x = sm.add_constant(x) *# Add a constant term to the regression model*
 model = sm.OLS(y, x).fit() *# Fit a linear regression model*
 slope = model.params[1] *# Get the slope of the line*
 intercept = model.params[0] *# Get the intercept of the line*
 r_squared = model.rsquared *# Get the R-squared value of the fitted line*
 y_pred = model.predict(x)
 rmse = np.sqrt(np.mean((y - y_pred) ** 2))
 **return** slope, intercept, r_squared, rmse, model

**def** plot_regression(slope, intercept, df, x_col, y_col, ax):
 x = df[x_col]
 y = df[y_col]
 ax.scatter(x, y)
 ax.plot(x, intercept + slope*x, color='#043e7d')
 **return**

**def** PR_multiple_old(dict):
 plt.rcParams['figure.figsize'] = [13, 13]
 plt.style.use("ggplot")
 fig, ax = plt.subplots(3,5, sharex=True)
 axe = ax.flatten()
 regression_results = []
 **for** i, key **in** enumerate(dict.keys()):
 slope, intercept, rs, rmse, model = linear_regression(dict.get(key), 'ELS_DAYS', 'IP_RIW')
 regression_results.append(
 {'Age Group': key,
 'Slope': slope,
 'Intercept': intercept,
 'R^2': rs,
 'RMSE': rmse,
 'No. of Observations': model.nobs,
 'F Value': model.fvalue,
 'P Value': model.f_pvalue})
 plot_regression(slope, intercept, dict.get(key), 'ELS_DAYS', 'IP_RIW', axe[i])
 axe[i].title.set_text(key)
 fig.supxlabel("Expected Length of Stay (Days)")
 fig.supylabel("Inpatient Resource Intensity Weight")
 fig.show()
 **return** regression_results

**def** PR_multiple(dict):
 plt.rcParams['figure.figsize'] = [13, 13]
 plt.style.use("ggplot")
 fig, ax = plt.subplots(3,5, sharex=True)
 axe = ax.flatten()
 regression_results = pd.DataFrame()
 **for** i, key **in** enumerate(dict.keys()):
 slope, intercept, rs, rmse, model = linear_regression(dict.get(key), 'ELS_DAYS', 'IP_RIW')
 regression_results = regression_results.append(
 {'Age Group': key,
 'Slope': slope,
 'Intercept': intercept,
 'R^2': rs,
 'R^2 Adjusted': model.rsquared_adj,
 'RMSE': rmse,
 'Residual Mean Squared Error': model.mse_resid,
 'No. of Observations': model.nobs,
 'F Value': model.fvalue,
 'P Value': model.f_pvalue}, ignore_index=True)
 plot_regression(slope, intercept, dict.get(key), 'ELS_DAYS', 'IP_RIW', axe[i])
 axe[i].title.set_text(key)
 fig.supxlabel("Expected Length of Stay (Days)")
 fig.supylabel("Inpatient Resource Intensity Weight")
 fig.show()
 **return** regression_results

PR_multiple(M1)

PR_multiple(M2)

PR_multiple(F1)

PR_multiple(F2)

LRT_multiples(dicts=[M1,M2,F1,F2], filename="linear_regression_results")

# Appendix D: Supplementary References

[1] Yue Yang, Yang Wu, Peikun Wang, and Jiali Xu. Stock price prediction based on xgboost and lightgbm, 2021. Copyright - © 2021. This work is licensed under https://creativecommons.org/

licenses/by/4.0/ (the “License”). Notwithstanding the ProQuest Terms and conditions, you may use this content in accordance with the terms of the License; Last updated- 2021-09-29.

[2] Chen Wang, Chengyuan Deng, and Suzhen Wang. Imbalance-xgboost: leveraging weighted and focal losses for binary label-imbalanced classification with xgboost. Pattern Recognition Letters, 136:190–197, 2020.

[3] Trevor Hastie, Robert Tibshirani, and Jerome Friedman. Random Forests, pages 587–604. Springer New York, New York, NY, 2009.

[4] Seyed Mehran Kazemi. Relational logistic regression. PhD thesis, University of British Columbia, 2014.

[5] S. Henrard, N. Speybroeck, and C. Hermans. Classification and regression tree analysis vs. multi-

variable linear and logistic regression methods as statistical tools for studying haemophilia. Haemophilia,

21(6):715–722, 2015

[6] Canadian Institute for Health Information. Discharge Abstract Database metadata (DAD) [Metadata]. Accessed March 14th, 2023.
